# NIBBS-Search for Fast and Accurate Prediction of Phenotype-Biased Metabolic Systems

**DOI:** 10.1371/journal.pcbi.1002490

**Published:** 2012-05-10

**Authors:** Matthew C. Schmidt, Andrea M. Rocha, Kanchana Padmanabhan, Yekaterina Shpanskaya, Jill Banfield, Kathleen Scott, James R. Mihelcic, Nagiza F. Samatova

**Affiliations:** 1 Department of Computer Science, North Carolina State University, Raleigh, North Carolina, United States of America; 2 Computer Science and Mathematics Division, Oak Ridge National Laboratory, Oak Ridge, Tennessee, United States of America; 3 Department of Civil and Environmental Engineering, University of South Florida, Tampa, Florida, United States of America; 4 Neuroscience Department, Duke University, Durham, North Carolina, United States of America; 5 Departments of Earth and Planetary Science, University of California, Berkeley, California, United States of America; 6 Environmental Science, Policy, & Management, University of California, Berkeley, California, United States of America; 7 Geochemistry Department, Lawrence Berkeley National Laboratory Earth Sciences Division, Berkeley, California, United States of America; 8 Department of Integrative Biology, University of South Florida, Tampa, Florida, United States of America; The Centre for Research and Technology, Hellas, Greece

## Abstract

Understanding of genotype-phenotype associations is important not only for furthering our knowledge on internal cellular processes, but also essential for providing the foundation necessary for genetic engineering of microorganisms for industrial use (e.g., production of bioenergy or biofuels). However, genotype-phenotype associations alone do not provide enough information to alter an organism's genome to either suppress or exhibit a phenotype. It is important to look at the phenotype-related genes in the context of the genome-scale network to understand how the genes interact with other genes in the organism. Identification of metabolic subsystems involved in the expression of the phenotype is one way of placing the phenotype-related genes in the context of the entire network. A metabolic system refers to a metabolic network subgraph; nodes are compounds and edges labels are the enzymes that catalyze the reaction. The metabolic subsystem could be part of a single metabolic pathway or span parts of multiple pathways. Arguably, comparative genome-scale metabolic network analysis is a promising strategy to identify these phenotype-related metabolic subsystems. Network Instance-Based Biased Subgraph Search (**NIBBS**) is a graph-theoretic method for genome-scale metabolic network comparative analysis that can identify *metabolic systems* that are statistically biased toward phenotype-expressing organismal networks. We set up experiments with target phenotypes like hydrogen production, TCA expression, and acid-tolerance. We show via extensive literature search that some of the resulting metabolic subsystems are indeed phenotype-related and formulate hypotheses for other systems in terms of their role in phenotype expression. NIBBS is also orders of magnitude faster than MULE, one of the most efficient maximal frequent subgraph mining algorithms that could be adjusted for this problem. Also, the set of phenotype-biased metabolic systems output by NIBBS comes very close to the set of phenotype-biased subgraphs output by an exact maximally-biased subgraph enumeration algorithm ( MBS-Enum ). The code (NIBBS and the module to visualize the identified subsystems) is available at http://freescience.org/cs/NIBBS.

## Author Summary

Genetic engineers often seek to modify the physical traits of microorganisms used in industrial processes in order to improve the efficiency of the overall process. The genes targeted for modification in these cases are typically identified by searching for genes whose presence in an organism is correlated with the presence of the physical trait. In the last few years, however, it has become understood that the physical traits of an organism are often the result of a coordinated set of interactions between multiple genes that make up a biological subsystem. This gives rise to a computational tractability problem, since the number of possible sets of genes is exponentially larger than the number of genes in an organism. Here, we use biological networks to limit the search space to sets of genes known to interact. The presence of the biological subsystems identified by this approach are shown to be significantly correlated to the presence of the phenotype. The results show that this framework can provide potential genetic targets for modifying the expression of a given phenotype.

## Introduction

Certain industrial processes, such as the production of hydrogen and ethanol, benefit from using prokaryotic or eukaryotic organisms to produce, reduce, and convert important chemical compounds [1], [2]. Bioengineers search for ways to modify phenotypic traits, or *phenotypes*, of these organisms to improve the overall process efficiency [3]. Modifications to the organism's phenotype are made through modifications to its genome. In order to obtain the desired changes in the organism's phenotype, engineers require a deciphering of which genes are related to the expression of the given phenotype, also known as *genotype-phenotype* associations [4], [5]. Unfortunately, such an understanding has not kept pace with the rate at which genes are discovered [6].

Uncovering genotype-phenotype associations could be greatly improved if organism's metabolic systems involved in the phenotype expression were understood [7]. These systems involve multiple metabolic reactions that are grouped into functionally-distinct modules called metabolic pathways [8]. Changes to the enzymes in these modules can affect the expression of the phenotype of interest. Thus, it is imperative to be able to identify all of the enzymes that make up a phenotype-related metabolic system.

The task of identifying a phenotype-related metabolic system consists of two main subtasks: determining the metabolic system and establishing that it is phenotype-related.

Understanding how a system has been evolutionarily conserved has been used as an approach to accomplish both tasks. If a set of interacting metabolic reactions are important for expressing the target phenotype, then there likely exists an evolutionary pressure to conserve the set as a whole, or to have them co-present together, in multiple organisms [9]. The assumed reason for this evolutionary pressure is that the set forms a metabolic system whose function is required by the organism and by its descendants [9].

This is the motivation behind network alignment and phylogenetic profiling approaches proposed to-date. The former [9]–[13] look for subgraphs that exist in metabolic networks of multiple organisms. The latter [4], [5], [14] seek to find genes or enzymes that are more likely to be present in phenotype-expressing organisms than in phenotype-non-expressing organisms due to an evolutionary pressure to conserve the phenotype-related enzymes [14].

However, neither network alignment nor phylogenetic profiling approaches can alone identify phenotype-related metabolic systems. Network alignment algorithms can identify metabolic systems present in all or most of a given set of organisms; such a set is typically small, e.g., less than 10 networks. However, even if the set of organisms exhibit a common phenotype, current network alignment approaches cannot distinguish phenotype-related metabolic systems from other common metabolic systems. Additionally, network alignment approaches would likely not identify a metabolic system if it is only common to a subset of the organisms being compared.

Phylogenetic profiling approaches can identify phenotype-related enzymes that are specific to phenotype-expressing organisms. However, it is possible that enzymes that are part of a phenotype-related metabolic system will not be specific to phenotype-expressing organisms; therefore, these approaches will likely miss them. Additionally, it would be computationally intractable to compare the presence of every possible set of enzymes to the presence of the phenotype.

**Figure 1:**
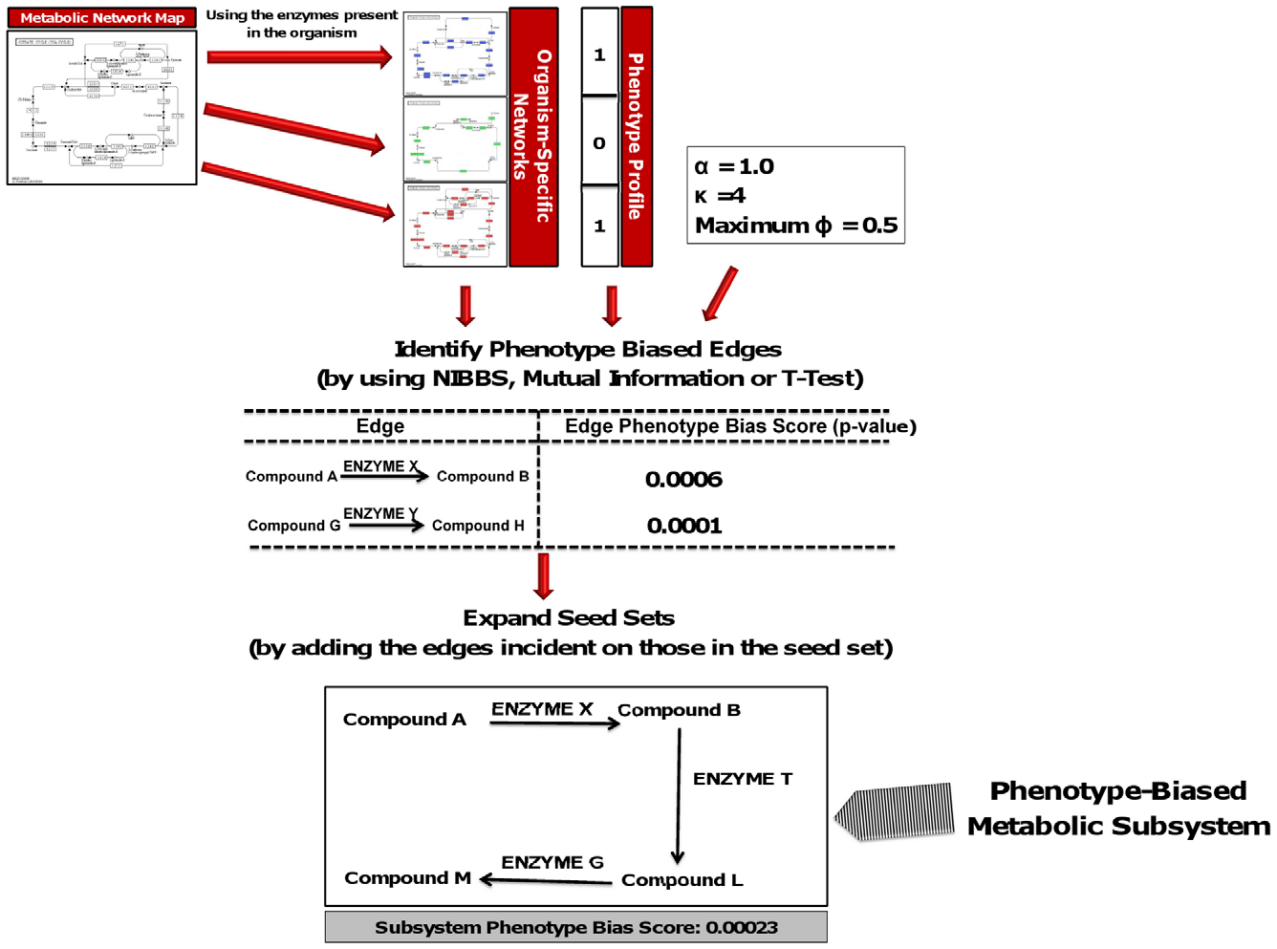
NIBBS methodology overview; The generic metabolic map is taken and converted into organism specific metabolic map, using the enzyme information of each organism. These networks along with the other algorithm parameters are used to first construct seed sets. These seed sets are then expanded into the final metabolic subsystem that is output by the algorithm. The details are provided in the *Methods* section.

In order to address these and other limitations of existing methods, in this paper, we introduce the Network Instance-Based Biased Subgraph Search ( NIBBS-Search ) algorithm (Figure 1) that enables *in silico*, fast, and accurate prediction of phenotype-related metabolic systems. The predictions arise from comparative analysis of multiple genome-scale metabolic networks. The approach is capable of predicting phenotype-related metabolic systems that are unlikely to be found by current *in silico* methods. These include but are not limited to metabolic systems that are specific to a subset of the phenotype-expressing organisms that may exhibit a sub-phenotype of the target phenotype (e.g., dark fermentative, light fermentative or bio-photolytic sub-phenotypes of biohydrogen production phenotype).

A network structure–a *maximally-&-phenotypically-biased* subgraph ( MPBS )–is introduced to model phenotype-related metabolic systems in a set of metabolic networks derived for dozens or even hundreds of organisms. To assess NIBBS-Search 's accuracy, we first present the Maximally-Biased Subgraph Enumeration ( MBS-Enum ) method that exactly enumerates all MPBS s in a given set of networks; these subgraphs are then used for comparison with the NIBBS-Search results. To overcome MBS-Enum's computational complexity, NIBBS-Search heuristically approximates the set of MPBS s in the set of networks. NIBBS-Search runs orders of magnitude faster than MBS-Enum , while identifying with high sensitivity subgraphs that are statistically significant approximations of the set of MPBS s. Also, the NIBBS-Search -predicted systems contain known phenotype-related enzymes and pathways, including those that only exist in a subset of the phenotype-expressing organisms.

## Results

### Overview of NIBBS

The NIBBS algorithm identifies *phenotypically-biased* edges from a metabolic map called the seeds and then expands each seed into a *maximally*, *phenotypically-biased* metabolic system. The method requires a set of organisms that express the phenotype of interest and ones that do not. A phenotype-profile vector is built for the organism set (see Figure 1). This organism phylogenetic profile vector and the organism-specific metabolic maps from the KEGG database [15]–[17] are provided as input to NIBBS. The organism-specific metabolic map is a graph, each edge corresponding to a metabolic reaction, substrates and products as its vertices at the two ends of the edge, and the edge label is the enzyme that catalyses the reaction. NIBBS as its first step identifies the phenotypically-biased edges called *seeds*. Informally, an edge is phenotypically-biased if it is present in a larger number of phenotype expressing organisms when compared to phenotype non-expressing organisms. The seed edges are then expanded into *maximally*, *phenotypically-biased* metabolic subsystems by the addition of other edges from the genome-scale metabolic map. The details are discussed in the *Methods* section.

### Materials

We identified both phenotype-expressing and phenotype non-expressing organisms via literature search. We primarily analyzed six main phenotypes, aerobic respiration, anaerobic respiration, TCA (citrate cycle) expression, rTCA (reverse TCA) expression, hydrogen production, and acid-tolerance. We also looked at three sub-phenotypes of hydrogen production: dark fermentation, light fermentation, and bio-photolysis. The summary of the organisms used for each experiment is listed in Table 1. We used the metabolic networks and enzyme lists available in the KEGG database [15]–[17]. The results for all the experiments are available as supplemental files in the website mentioned in the abstract.

**Table 1:** Number of organisms per phenotype.

Phenotype	Phenotype Expressing	Phenotype Non-expressing
Aerobic	33	54
Anaerobic	54	33
TCA	15	6
rTCA	6	15
Hydrogen Production	17	11
Dark Fermentation	8	11
Light Fermentation	5	11
Bio-photolysis	4	11

#### Organism selection process

For this study, we selected sets of completely sequenced genomes representative of both phenotype and phenotype non-expressing microorganisms. Genomic information for each organism within the dataset was obtained from the KEGG database and then incorporated into the NIBBS search algorithm. For each phenotype, an extensive literature review of published papers and microbial databases was conducted to identify representative microorganisms. Examples of microbial databases searched include the Department of Energy's (DOE's) Joint Genome Institute (JGI) and the National Center for Biotechnology Information (NCBI) database. To ensure our results captured biochemical processes related to the phenotype in question and not of a specific genus, each data set contained a diverse group of microorganisms representative of various taxa. The only exception is the acid-tolerant phenotype. In this case, the organism list consisted mainly of Firmicutes. The entire list of organisms used in the various experiments is available in Table S1.

In the following sections, we demonstrate the applicability of the NIBBS-Search algorithm to identify phenotype-related metabolic processes involved in the production of biological hydrogen. In addition to hydrogen production, we included the acid-tolerant phenotype to our studies to identify potential acid-tolerant response systems. For hydrogen producers, the presence of these systems is important in respect to acidogenesis. During acidogenesis, organic acids e.g., butyrate and acetate are produced, resulting in the lowering of pH within the environment [18]. Without a response system, microorganisms will shift their metabolic routes from the production of acids and hydrogen to the production of solvents [19].

To further validate the NIBBS-Search algorithm's ability to predict phenotype-related metabolic processes (e.g., enzymes and subpathways), we selected the aerobic, anaerobic, TCA, and rTCA expressing phenotypes. The aerobic and anaerobic phenotypes are both well-characterized, thus we can validate through literature known phenotype-related biochemical processes. The TCA and rTCA expressing phenoyptes were selected to demonstrate the ability of the NIBBS-Search algorithm to identify phenotype-related enzymes within pathways that contain common enzymes. While these two studies do not directly relate to hydrogen production, they do serve to demonstrate the sensitivity of the algorithm.

### Bio-hydrogen Production

#### Hydrogen-production phenotype overview

Production of biological hydrogen is a potentially important sustainable technology for generation of alternative energy and fuels. The continuously growing number of naturally occurring microorganisms able to utilize various metabolic processes and organic substrates to generate hydrogen gas makes bio-hydrogen production a feasible option for development of bio-energy technologies[20]–[22]. One such technology of particular interest is the utilization of wastewater and waste materials for bio-hydrogen production[21], [23]. In these systems waste materials, such as food waste, contain numerous organic compounds that can be utilized by hydrogen producers for microbial growth and production of hydrogen gas [21].

#### Hydrogen-production types: Dark, Light, and Bio-photolysis

To generate hydrogen gas, hydrogen producers utilize one of three main metabolic processes. They are light fermentation, dark fermentation of organic matter, and decomposition of water by photosynthesizing microorganisms (bio-photolysis) [18], [20], [24]. A summary of these metabolic processes is provided below since they have been outlined in detail elsewhere [23], [25], [26]. Bio-photolysis, photosynthetic organisms can breakdown water molecules into hydrogen gas and oxygen [18], [20], [27], [28]. Production of hydrogen through this process can be carried out either directly by exposure to solar radiation or indirectly under dark (fermenting) conditions [29]. In light fermentation, organisms utilize simple organic compounds as a carbon source (e.g., glucose and sucrose) and a light source (e.g., sunlight) to generate hydrogen [18], [28], [30]. Dark fermentative bacteria differ from the previous two hydrogen-producing methods in that hydrogen evolving reactions are carried out without light energy by a number of heterotrophic bacteria [20], [31]. In this process, hydrogen is produced from dark fermentation reactions when organic substrates are utilized by heterotrophic bacteria as both the carbon and energy source for heterotrophic growth [20], [31]. Of the hydrogen-producing organisms associated with wastewater and waste materials, a majority appear to utilize dark fermentation metabolic processes to produce hydrogen. As such, in this paper, we focus on dark fermentative hydrogen production. The NIBBS results are available in Tables S2, S3, S4, S5, S6, S7, S8, S9, S10, S11.

#### Dark fermentation

**Figure 2:**
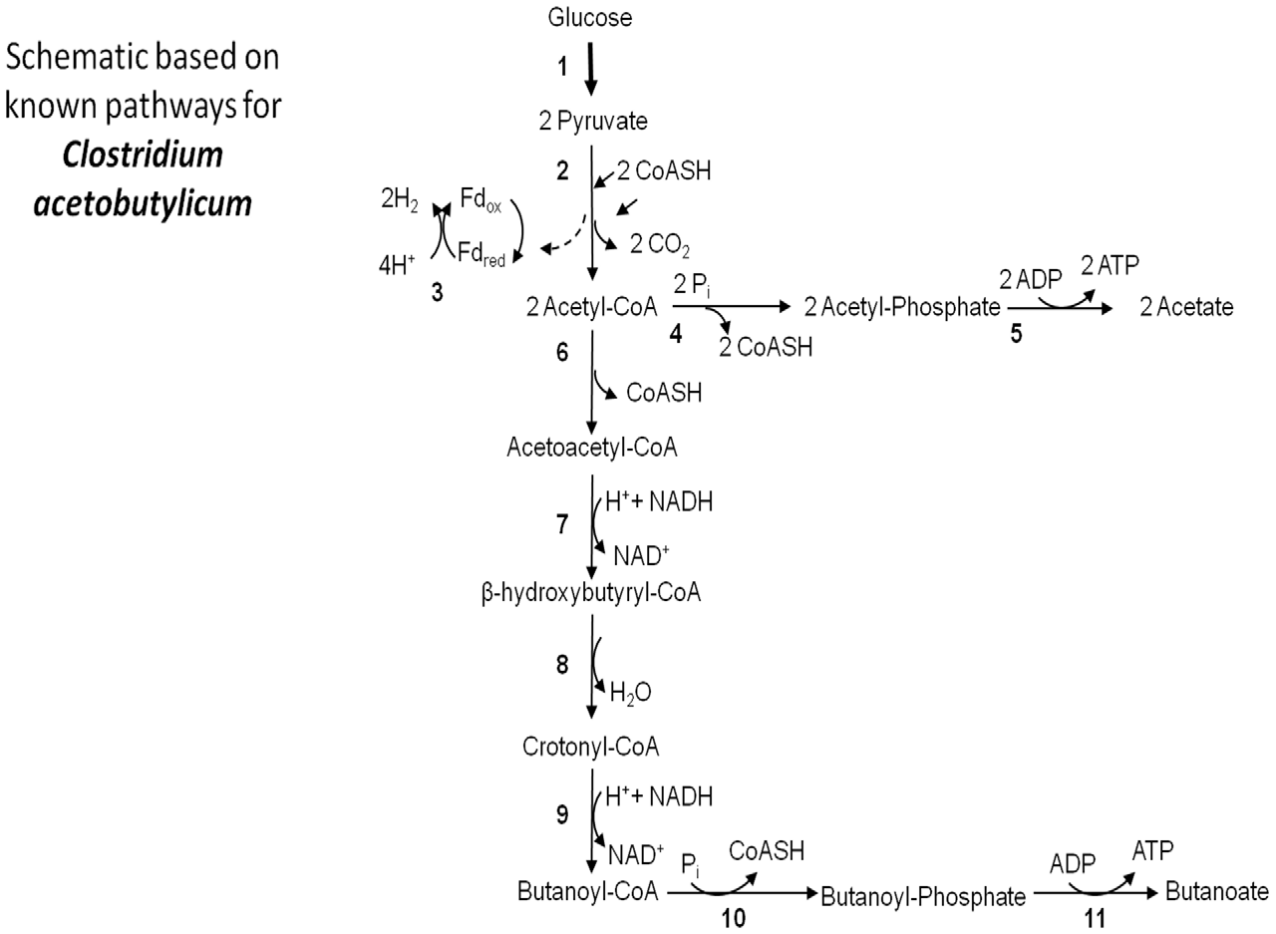
Schematic of key metabolic pathways for hydrogen production in *Clostridium acetobutylicum*. Arrows with larger width indicate a series of reactions. Arrows with narrow width indicate individual reactions. Enzymes: 1, glycolytic enzymes; 2, pyruvate ferredoxin oxidoreductase (E.C. 1.2.7.1); 3, hydrogenase (E.C.1.12.7.2); 4, phosphotransacetylase (E.C. 2.3.1.8); 5, acetate kinase (E.C. 2.7.2.1); 6, acetyl-CoA acetyltransferase (thiolase) (E.C. 2.3.1.9); 7, 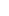-hydroxybutyryl-CoA dehydrogenase (E.C. 1.1.1.157); 8, crotonase (E.C. 4.2.1.55); 9, butyryl-CoA dehydrogenase (E.C. 1.3.99.2); 10, phosphotransbutyrylase (E.C.2.3.1.19); 11, butyrate kinase (E.C. 2.7.2.7). Abbreviations: Ferredoxin (Fd); Coenzyme A (CoASH).

Using *Clostridium acetobutylicum* as a model organism for dark fermenting hydrogen producers, the key metabolic pathways for hydrogen production, shown in Figure 2, were examined for the presence or absence of enzymes involved in each pathway. Analysis was conducted using predicted enzymes by the NIBBS method using the seed set generation process and the knowledge priors provided by the Student's T-Test. The two pathways, acetate and butanoate (i.e., butyrate), were selected as specific pathways for hydrogen production based on their potential hydrogen yield.

Table 2 shows that within the acetate pathway, NIBBS identified all of the constituent enzymes, pyruvate formate lyase (E.C. 2.3.1.54), acetate kinase (E.C. 2.7.2.1), and phosphotransacetylase (E.C. 2.3.1.8), as present within *C. acetobutylicum*. Whereas the T-Test only identified E.C. 2.3.1.8, all seven enzymes active in the butyrate pathway were found by the NIBBS method. The component enzymes for this pathway are butyryl-CoA dehydrogenase (E.C. 1.3.99.2), phosphate butyryltransferase (E.C. 2.3.1.19), butyrate kinase (E.C. 2.7.2.7), 3-hydroxybutyryl-CoA dehydrogenase (E.C. 1.1.1.157), acetyl-CoA C-acetyltransferase (E.C. 2.3.1.9), pyruvate formate lyase (E.C. 2.3.1.54), and crotonase (E.C. 4.2.1.55). Among these, only three were found by the T-Test.

**Table 2:** Hydrogen-related enzymes detected by different methods.

Pathway Name	EC Number	Enzyme Name	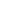	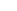	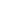
Acetate	2.7.2.1	acetate kinase			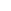
	2.3.1.8	phosphotransacetylase	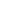		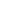
	4.2.1.55	crotonase	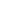		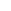
	2.3.1.54	butyryl-CoA dehydrogenase			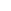
Butyrate	1.3.99.2	butyryl-CoA dehydrogenase			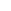
	1.3.99.2	butyryl-CoA dehydrogenase	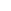		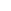
	2.7.2.7	butyrate kinase			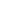
	1.1.1.157	3-hydroxybutyryl-CoA dehydrogenase	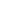		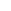
	2.3.1.19	phosphate butyryltransferase			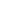
	2.3.1.9	acetyl-CoA C-acetyl-transferase			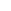
	2.3.1.54	pyruvate formate lyase	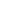		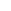
Formate	1.12.1.2	formate dehydrogenase	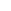		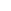
	1.2.7.1	pyruvate formate lyase			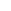
	1.12.7.2	ferrodoxin hydrogenase			

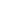
: Students' 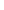-test; 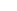: Mutual Information; 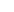: NIBBS-Search .

**Figure 3:**
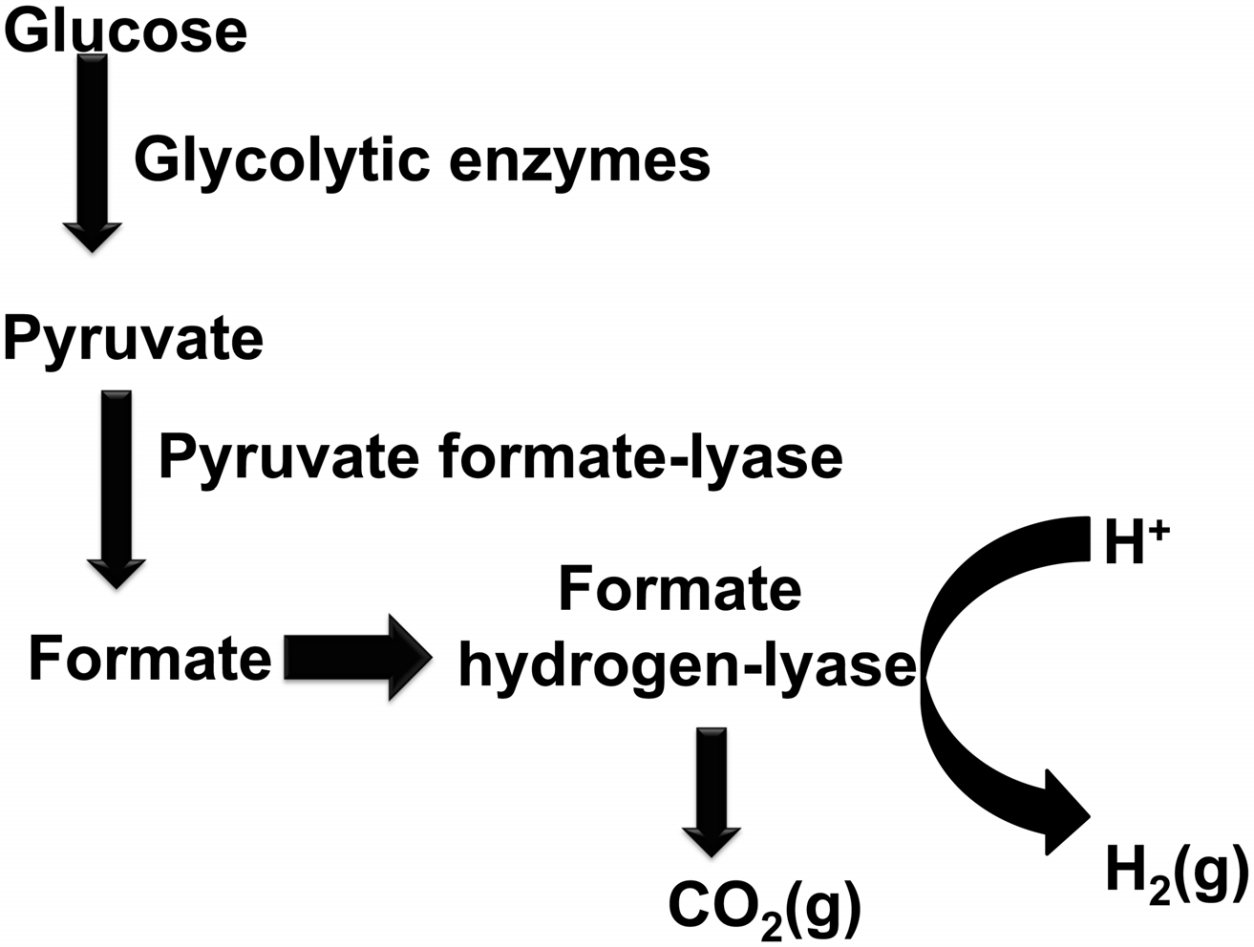
General overview of hydrogen production through the formate pathway.

In addition to the above pathways, the formate pathway was also reviewed. A general overview of formate production is shown in Figure 3. While it is not reported in the literature that *C. acetobutylicum* utilizes a formate pathway, it is possible that *C.acetobutylicum* may contain genes encoding some enzymes necessary for formate production. Of the three key enzymes described in Figure 3, NIBBS was able to identify only two of them. These are pyruvate formate lyase (E.C. 2.3.1.54) and formate dehydrogenase (E.C. 1.12.1.2). The second enzyme that along with formate dehydrogenase forms the formate hydrogen lyase complex is ferredoxin hydrogenase (E.C. 1.12.7.2) [32]. This enzyme is common in many organisms and is not phenotype-specific toward dark fermentation.

Other enzymes identified using the NIBBS algorithm, include those involved in glycolysis and nitrogen fixation. In this study, a large number of enzymes involved in glycolysis were predicted as conserved across hydrogen producing organisms but not conserved across hydrogen non-producing organisms [33]. This is mostly a result of the ability of the dark fermentative organisms to utilize organic compounds, such as glucose, for their carbon source. In terms of hydrogen production, glycolysis is a preliminary step needed for acetate or butyrate production as was depicted previously in Figure 2. In addition, glycolysis provides the energy sources necessary for biological hydrogen production to occur.

#### Pathways related to dark fermentative hydrogen production

From analysis of the enzymes identified in the previous section, the NIBBS algorithm was able to identify the most relevant metabolic pathways for dark fermentative hydrogen production. While these pathways are important for hydrogen production, additional metabolic pathways present within organisms may also play an important role in impacting hydrogen yields.

Using NIBBS, the following pathways were identified as top ranking metabolic pathways (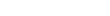) for *C. acetobutylicum* (Table S12) with respect to dark fermentative hydrogen production using the hypergeometric test (described in the Method section). They are: fatty acid biosynthesis (KEGG pathway ID ec00061), purine metabolism (KEGG pathway ID ec00230), arginine and proline metabolism (KEGG pathway ID ec00330), and cysteine and methionine metabolism (KEGG pathway ID ec00270). An overview of these pathways and their relation to hydrogen production is presented in the following sections. A complete listing of the pathways with their rankings is presented in Table 3.

**Table 3:** List of top ranking pathways and their enrichment score for the phenotype dark fermentative hydrogen production.

Pathway ID	Pathway Name	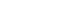
cac00061	Fatty acid biosynthesis	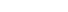
cac00230	Purine metabolism	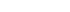
cac00330	Arginine and proline metabolism	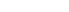
cac00520	Amino sugar and nucleotide sugar metabolism	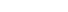
cac00270	Cysteine and methionine metabolism	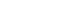
cac00030	Pentose phosphate pathway	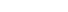
cac00040	Pentose and glucuronate interconversions	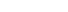
cac00400	Phenylalanine, tyrosine and tryptophan biosynthesis	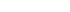
cac00051	Fructose and mannose metabolism	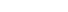
cac00260	Glycine, serine and threonine metabolism	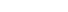
cac00860	Porphyrin and chlorophyll metabolism	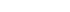
cac00250	Alanine, aspartate and glutamate metabolism	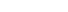
cac00920	Sulfur metabolism	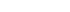
cac00500	Starch and sucrose metabolism	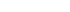
cac00480	Glutathione metabolism	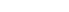
cac00300	Lysine biosynthesis	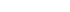
cac00910	Nitrogen metabolism	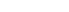
cac00010	Glycolysis & Gluconeogenesis	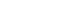
cac00052	Galactose metabolism	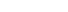

*Fatty Acid Biosynthesis:* Fatty acids are methylene carbon chains with a carboxyl group that are generally associated with the formation of structural membranes and maintenance of the membrane's fluidity [34]. Within bacteria fatty acids may be present in different forms such as branched, long chain, short chain fatty acids, volatile, or hydroxylated [34]. Formation or synthesis of fatty acids is generally initiated through the carboxylation of the acetyl-CoA [35]. In dark fermentative bacteria, such as *C. acetobutylicum*, acetyl-CoA is an important intermediary that leads to formation of acetate, butyrate, solvents, and fatty acids. As such, redirection of metabolic pathways away from fatty acid formation and towards acidogenesis (e.g., acetate formation) is vital for enhanced hydrogen production.

Analysis of results showed that fatty acid biosynthesis was the highest ranking metabolic pathway for *C. acetobutylicum* in both the phenotype and its sub-phenotype–hydrogen producing organisms and dark fermentative hydrogen producing organisms, respectively. The presence of this pathway in both categories suggests that fatty acid biosynthesis may play a key role in regulating metabolic routes for hydrogen formation, specifically, in dark fermentation. Findings in this study are similar to previous reports on the role of fatty acids in acetate and butyrate formation. In a study by Huang *et al.* [36], the presence of short-chained fatty acids during acidogenesis was linked to initiation of solventogenesis to form butanol and acetone in fermenting bacteria [36]. This is a resultant of fatty acid build-up within the cells. As the short chain fatty acids accumulate, bacterial cells form a transmembrane pH gradient leading to induction of solvent production [36].

*Purine Metabolism:* Purines are nucleotide bases that can be found either in free forms or attached to ribose 5-phosphate to form nucleotides and nucleic acids [34]. Organisms may synthesize purine nucleotides for use in the structural make-up of nucleic acids or use in ATP metabolism [37]. During purine synthesis, amino acid donors are utilized to form purine rings and other purine structural components. Examples of amino acid donors include glutamine and aspartic acid [34]. In free form, purine nucleotide bases are harmful and toxic to the organisms, so they must be removed or transformed to non-toxic compounds. As such, many organisms have mechanisms to anaerobically degrade purine compounds through fermentation of xanthine into intermediates, which could potentially form acetate and formate [38]. One such organism capable of purine degradation is *Clostridium ljungdahlii* [39]. In *C. ljungdahlii*, purine compounds are degraded to form intermediates, such as glycine and betaine. These intermediates in turn are reduced, resulting in acetate formation by the enzyme acetate kinase [39], [40].

Depending on the respiration requirement of the organisms (e.g., aerobic versus anaerobic), the degradation pathway used by microorganisms will vary. In our study, we selected dark fermentative hydrogen producers and within this phenotype, we include both facultative anaerobic and anaerobic bacteria. As such, an extensive review of metabolic reactions is necessary to determine which degradation pathways, if any are utilized. However, based on the high ranking of this pathway in our study for *C. acetobutylicum*, we can predict that purine metabolism (degradation and synthesis) plays a minor role in generation of acetate in dark fermentative bacteria.

*Arginine and Proline Metabolism:* L-Proline and L-arginine are two amino acids commonly found within both eukaryotic and prokaryotic organisms [41], [42]. In bacterial cells, L-proline is synthesized from L-glutamate by glutamate kinase [42], [43]. In addition to biosynthesis of proline, some bacteria have been reported to take up and utilize proline as either a carbon or nitrogen source for metabolic growth [44]. In *Escherichia coli*, proline and proline betaine have been linked to increased osmotolerance and protection in cells [45]. Such protection would be beneficial in dark fermentation species for microbial response to induce water stress.

L-arginine is also an important precursor in nitrogen metabolism and protein synthesis in bacterial cells [41]. It can be metabolized by cells to produce other amino acids, including proline, or utilized by the cell as either a carbon or nitrogen source. In addition, L-arginine may serve as an energy source for anaerobic bacteria. This is done through ATP production from L-arginine in the arginine deiminase pathway [41]. L-arginine biosynthesis occurs similar to L-proline in requiring L-glutamate as a precursor to biosynthesis. In this process, L-glutamate is deaminated through the enzyme glutamate dehydrogenase.

In this study, arginine and proline metabolism was identified as a potentially important pathway for *C. acetobutylicum* with respect to both hydrogen producing organisms and the sub-phenotype, dark fermentative hydrogen production. In addition to identifying arginine and proline metabolism in individual species, evaluation of hydrogen production related enzymes shows that this pathway is significant and likely related to hydrogen production.

*Cysteine and Methionine Metabolism:* Methionine is a sulfur-containing amino acid that is used for biosynthesis of cysteine [46]. In general, most organisms can either take-up methionine or synthesize it to form other amino acids and help initiate protein synthesis [47]. Cysteine is another sulfur-containing amino acid important for the production of glutathione, a compound that aids in protecting the cell from oxidative stress [47], [48]. In hydrogen producing organisms, cysteine ligands and residues play an important role in the structure of [Fe-S] clusters and hydrogenase enzymes [47], [49], [50]. Additionally, cysteine ligands aid in the binding of [Fe-S] clusters together with nitrogenase enzymes [51]. Nitrogenase enzymes are typically found in nitrogen fixing bacteria and are considered key enzymes to hydrogen production in light fermentative bacteria [52]. However, studies on nitrogen fixation have found that many dark fermentative species, such as *Clostridium*, are capable of utilizing nitrogenase enzymes [53]. However, in this study we do not consider hydrogen production through nitrogenase as a key metabolic route. This is mainly due to the energy expense needed for nitrogen-fixation by organisms such as *C. acetobutylicum*.

The role of cysteine and methionine in formation of [Fe-S] clusters for both hydrogenase and nitrogenase activity demonstrates the relationship of this cysteine and methionine metabolism in hydrogen producing organisms. From the analysis, this KEGG pathway was predicted as a significant metabolic route in both the *C. acetobutylicum* and in the set of organisms expressing the phenotype hydrogen production (see Table 3).

### Acid-tolerance

In order to predict enzymes related to a microorganism's ability to tolerate low pH conditions, ten acid-tolerant organisms and eight alkaliphiles were analyzed using the NIBBS algorithm (Table S13). Analysis of the NIBBS enzymes shows that 73% acid-tolerant enzymes were recalled, when acid-tolerant organisms were used as positive instance. NIBBS enzymes predicted 164 enzymes, while the Student's T-Test identified only 17 as phenotype-related. Enzymes identified by the Student's T-Test and missed by NIBBS included enzymes involved in central metabolism, amino acid metabolism, and lactic acid metabolism.

#### Acid-tolerant enzymes

To identify acid-tolerant enzymes, *C. acetobutylicum* was used as our model organism. In many fermentative, hydrogen producing experiments and in natural systems, acetogenic *Clostridium* species are often present. Review of the literature indicated that *C. acetobutylicum* and many other hydrogen producing species can tolerate and maintain hydrogen production in acidic pH ranging from 4.5 to 6 [36]. To survive, these organisms have developed metabolic and cellular acid-tolerance response (ATR) systems to protect themselves when exposed to acid environments [54]. While a few acid-tolerant or resistant systems have been described in organisms such as *Lactobacilli*, the little is known about metabolic pathways involved in acid-tolerance, particularly in *Clostridium* species.

Analysis of the predicted enzymes for *C. acetobutylicum* did not reveal a distinct acid resistance metabolic system. However, review of the predicted enzymes across other hydrogen producers revealed the potential of an acid resistance system. Identified enzymes included glutamate decarboxylase (E.C. 4.1.1.15; Gad), a known enzyme involved in acid-resistance in some microorganisms including *Clostridium perfringens*, a known hydrogen producer. In *Escherichia coli*, *C. perfringens*, and some *Lactobacilli* the internal pH can be neutralized by a decarboxylase system–glutamate and arginine decarboxylase [54]–[56]. In *Lactobacilli*, glutamate decarboxylase converts glutamate to 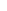-amino butyric acid (GABA), which is quickly removed and replaced by another glutamate molecule [54]. While glutamate decarboxylase plays a vital role in this decarboxylase system, other proteins and antiporters are required for neutralization of the internal pH to occur.

Glutamate decarboxylase was only present in three of our ten acid-tolerant organisms (Table S14). They are *Lactobacillus plantarum* JDM1, *Lactobacillus plantarum* WCFS1, and *Clostridium perfringens* ATCC 13124. Prediction of glutamate decarboxylase by NIBBS was due to the presence of the enzyme in a small subset of organisms within our dataset and the absence of the enzyme in phenotype non-expressing organisms. Based on the absence of glutamate decarboxylase in many of our organisms, including hydrogen producing *C. acetobutylicum* and *C. beijerinckii*, we can classify glutamate decarboxylase as not specific for, but rather related to acid-tolerance. The presence in *C. perfringens* and absence within other *Clostridium* species do not necessarily indicate that *C. acetobutylicum* is not capable of similar mechanisms. In fact, incorporation of a decarboxylase system similar to that of *C. perfringens* and *L. plantarum* into hydrogen producers, such as *C. acetobutylicum* may be necessary to maintain hydrogen production and acidogenesis.

#### Acid-tolerant pathways

Metabolic pathways related to expression of acid-tolerance, vary across organisms and sub-sets of organisms, as shown by analysis of phenotype-related enzymes. This is particularly true between Gram negative and Gram positive organisms [54], which contain different response mechanisms for acid exposure. In this study, acid-tolerant organisms selected consisted mainly of Gram positive, acid-tolerant bacteria from the phylum *Firmicutes*. As such, results reflect metabolic pathways present to a small group of bacteria capable of acid-tolerance rather than across a diverse set of organisms capable of expressing the acid-tolerant phenotype.

Using the NIBBS-Search algorithm, seven enriched pathways (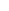-value
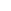
0.05) (Table S15) were identified using the hypergeometric test (described in the Method section). Of these pathways, the following metabolic pathways were predicted as top ranking with respect to acid-tolerance based on enzyme enrichment. They are purine metabolism (KEGG pathway ID ec00230) and arginine and proline metabolism (KEGG pathway ID ec00330). A list of pathways and their enrichment scores are presented in Table 4. Since the basic role of purine metabolism and arginine and proline metabolism was described in detail in the previous section, we will focus mainly on the relationship of the pathway with respect to acid-tolerance.

**Table 4:** List of top ranking pathways and their enrichment score for the phenotype acid-tolerance.

Pathway ID	Pathway Name	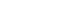
cac00230	Purine metabolism	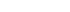
cac00330	Arginine and proline metabolism	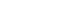
cac00520	Amino sugar and nucleotide sugar metabolism	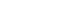
cac00260	Glycine, serine and threonine metabolism	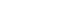
cac00270	Cysteine and methionine metabolism	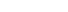
cac00400	Phenylalanine, tyrosine and tryptophan biosynthesis	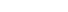
cac00240	Pyrimidine metabolism	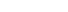
cac00860	Porphyrin and chlorophyll metabolism	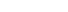
cac00760	Nicotinate and nicotinamide metabolism	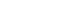
cac00500	Starch and sucrose metabolism	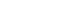
cac00040	Pentose and glucuronate interconversions	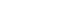
cac00561	Glycerolipid metabolism	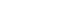
cac00051	Fructose and mannose metabolism	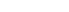

*Purine Metabolism:* NIBBS-Search algorithm predicted purine metabolism as a potentially significant pathway for organisms expressing acid-tolerance.

Purine metabolism encompasses biosynthesis, degradation, and salvage of purines within microorganisms. Together these pathways are necessary for survival and growth of organisms. Purines, along with pyrimidines, make-up vital components of nucleic acids (e.g., DNA and RNA), and are involved in synthesis of many vitamins and coenzymes (e.g., ATP) [34]. As such, the high ranking of purine metabolism is likely a result of its role in nucleic acid synthesis (and growth) rather than specificity to the acid-tolerant phenotype. However individual enzymes present within purine metabolism may play a role in maintaining purine and nucleic acids during periods of acid stress. In fact, studies evaluating acid resistance, have realized the potential of purine genes, *deoB* and *guaA*, that encode for phosphopentomutase and GMP synthase, respectively, in assisting with acid-tolerance [54]. Proteins associated with these genes are involved in the salvage pathway. In some *Lactobacillus* species, organisms can utilize nucleobases, such as guanine and adenine, generated during DNA and RNA degradation to synthesize nucleotides [57]. The salvage of these purine nucleobases is particularly important during dark fermentative hydrogen production when organic acid (e.g., butyrate) accumulation lowers pH in the medium. If the internal pH value is not regulated and decreases, DNA and purine bases present are subject to degradation [56]. The presence of salvage pathway enzymes, such as adenosine deaminase, allows organisms to utilize the degraded bases to regenerate nucleotides and nucleic acids. Therefore, we predict that sub-pathways within the purine salvage are related to expression of acid-tolerance and resistance. Experimental analysis is needed to determine the exact role of purine salvage in bacterial response to low pH.

*Arginine and Proline Metabolism:* In hydrogen producing organisms, decarboxylation and deamination of amino acids (e.g., arginine) have been linked to osmotolerance and protection of cells in the presence of environmental stress [54]. One amino acid in particular is arginine. While arginine can be an important source of nitrogen and energy for bacteria, it is also considered an alkaline amino acid, thus making it an important component in combating acid stress. One mechanism involving arginine is decarboxylation of glutamate and arginine in *Lactobacilli*. In this process, arginine is decarboxylated, then the decarboxylated product is removed and another arginine product is transported into the cell [54]. Another mechanism is the arginine deiminase pathway (ADI). This pathway is responsible for the conversion of arginine to orthine, ammonium, and carbon dioxide. The ammonium produced is then used to increase the internal pH [54].

From the predicted NIBBS results, the presence of the ADI or decarboxylation pathways was not predicted in our model organism, *C. acetobutylicum*. However, key enzymes involved in these pathways for *C. perfringens* were shown as present, thus suggesting these pathways may be utilized by this organism in response to acid stress. For the first pathway, the NIBBS algorithm was only able to predict the presence of glutamate decarboxylase (E.C.4.1.1.15) and did not identify arginine decarboxylase. This suggests that *C. perfringens* may not utilize this route for acid-tolerance.

For the ADI pathway, only two of the three essential enzymes associated with this pathway were identified. They are arginine deiminase (E.C. 3.5.3.6) and ornithine transcarbamylase (E.C. 2.1.3.3). In addition, we noted the presence of agmatine deiminase (E.C 3.5.3.12), an enzyme responsible for conversion of agmatine to N-carbamoylputrescine and ammonia. Based on the presence of agmatine deiminase, we predict that *C. pefringens* may utilize this enzyme in arginine metabolism in response to acid stress. While it does not appear that *C. acetobutylicum* utilizes these two pathways, there have been reports suggesting that it is capable of utilizing similar mechanisms through activation of homologous genes [56]. However, review of these types of genes has not been well characterized to date. As such, analysis of genes present in the hydrogen producing *C. pefringens* can be used to provide clues to expression of acid-tolerance.

### Methodology Validation

Two experiments were performed to measure the ability of the NIBBS algorithm to identify enzymes and potential subpathways related to organisms capable of expressing specific pathways. In order to assess the ability of both approaches to identify phenotype-related enzymes, 36 aerobic organisms and 36 anaerobic organisms were selected. Analysis of the NIBBS enzymes shows 86% and 75% recall, respectively, when one or the other are used as positive instances. The results showed that NIBBS enzymes for aerobic respiration contained 261 enzymes and for anaerobic respiration contained 93 enzymes, while the Student's T-Test identified 131 enzymes for aerobic respiration and 64 enzymes for anaerobic respiration.

Examination of the enzymes found by the Student's T-Test but missed by NIBBS-Search shows that they are typically present in most of the phenotype-expressing and non-expressing organisms. The reason some enzymes are identified as phenotype-related by the statistical analysis is due to the fact that they typically have a higher copy number in phenotype-expressing organisms. Since NIBBS-Search uses binary data (i.e., whether at least one copy of the enzyme is present in the organism), these enzymes are not identified by NIBBS-Search as biased. In addition, because the NIBBS algorithm does not rely on the enzyme distributions across entire sets of organisms, it is capable of identifying subgroups of organisms among the list of given species. As such, it is not expected that NIBBS will contain identical sets of enzymes as those identified with the Student's T-Test approach.

#### Enzymes predicted by NIBSS for aerobic and anaerobic organisms

Evaluation of phenotype-related enzymes identified for aerobic organisms show that the NIBBS algorithm was able to discover a small set of known enzymes associated with pathways commonly associated with the phenotypes of aerobic and autotrophic carbon fixation. In Table 5, enzymes typically associated with aerobic organisms consisted of enzymes that make up components of the TCA cycle and the glyoxylate bypass.

**Table 5:** Known aerobic related enzymes that make up the TCA cycle and the glyoxylate bypass that are present (+) or absent (−) in the data set identified by the NIBBS algorithm and T-Test approach.

EC Number	Enzyme Name	Pathway	NIBBS	T-Test
2.3.3.1	citrate (Si)-synthase	TCA, glyoxylate bypass	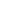	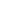
1.2.4.2	oxoglutarate dehydrogenase (succinyl-transferring)	TCA	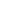	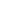
1.3.99.1	succinate dehydrogenase	TCA	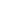	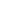
1.1.1.37	malate dehydrogenase	TCA, glyoxylate bypass	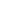	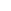
4.1.3.1	isocitrate lyase	glyoxylate bypass	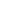	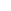
2.3.3.9	malate synthase	glyoxylate bypass	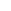	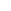
6.2.1.5	succinate–CoA ligase (ADP-forming)	TCA	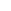	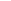
4.2.1.2	fumarate hydratase	TCA	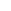	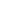
1.1.1.42	isocitrate dehydrogenase (NADP+)	TCA	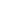	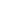
4.2.1.3	aconitate hydratase	TCA, glyoxylate bypass	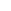	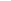

Other enzymes identified as phenotype-related are present due to phenotype associations with sub-groups of organisms in our dataset. These include organisms with similar fatty acid metabolism, amino acid metabolism, and photosynthetic organisms. Enzymes predicted as related to anaerobic organisms included 2-oxoglutarate synthase and ATP-dependent citrate lyase, which are related to the reductive TCA (rTCA) cycle (Table S16). The enzyme results associated with the anaerobic organisms are counter intuitive since rTCA is an autotrophic carbon fixation pathway and not associated with the anaerobic phenotype. The finding of rTCA-related enzymes is likely related to a subset of organisms or subphenotype present in the dataset.

### TCA vs. rTCA Pathway

Due to the ability of the NIBBS-Search algorithm to predict phenotype-related enzymes through the prediction of phenotype-related metabolic systems, the algorithm is capable of distinguishing between pathways that contain common enzymes. To demonstrate this feature of NIBBS-Search, two experiments were conducted comparing the two well-characterized metabolic networks, tricarboxylic acid (TCA) cycle and the reverse TCA (rTCA) cycle.

Sets of organisms known to utilize the TCA and rTCA cycle were selected and analyzed (Table S16). Selection of the two metabolic systems was due to the ability of these pathways to utilize the same set of metabolites and have common enzymes.

Using sixteen organisms that utilize the TCA cycle and six organisms that utilize the rTCA cycle, NIBBS algorithm was able to identify all but one TCA enzyme, malate dehydrogenase (EC 1.1.1.37), among the top ranking systems (Table S17). Malate dehydrogenase is part of another system which also includes seven of the eight TCA enzymes (isocitrate dehydrogenase is not included). All eight of the TCA enzymes are, therefore, part of at least one statistically significant system identified in the TCA experiment. To ensure the sensitivity of the algorithm to identifying key enzymes characteristic for each pathway, we reviewed the results to determine if key rTCA enzymes were present in any of the positive instances. In this study, we did not identify any of the three key enzymes unique to rTCA and this suggests that the NIBBS algorithm was able to properly predict the TCA pathway for phenotype-expressing organisms.

Similar results are obtained in the rTCA experiment (Table S18), when rTCA-utilizing organisms are used as positive instances. A top ranking system identified in the rTCA experiment contains seven of the eight rTCA enzymes, including all the five enzymes that the rTCA cycle shares with the TCA cycle (Table S16). The rTCA-related enzyme, fumarate reductase (EC 1.3.1.6) was not indicated as present in any system identified in the rTCA experiment.

In the rTCA experiment, systems identified by NIBBS include two enzymes, citrate synthase (EC 2.3.3.1) and succinate dehydrogenase (EC 1.3.99.1) that are typically associated with the TCA pathway [58]. This is because these two enzymes are not only present in all of the rTCA expressing organisms in the experiment but also in most, if not all, of the TCA expressing organisms in the experiment. This makes them likely to be included in the set of expansion edges, since they do not decrease the–value of the system.

The presence of these TCA-related enzymes in rTCA related systems does not indicate an additional functionality, but rather that succinate dehydrogenase found by KEGG might actually be acting as a fumarate reductase. Being that the two enzymes are evolutionarily related to each other, fumarate reductase and succinate dehydrogenase are difficult to distinguish based on sequence alone [59].

### Comparison with Related Methods

#### Comparison with Slonim *et al* [4] method

To assess the ability of NIBBS algorithm to identity phenotype-related enzymes and pathways, we compared NIBB's seed generation to the Mutual Information method described by [4] (Table 2). Using seed enzymes presented for hydrogen production versus hydrogen non-production, we find that Mutual Information only identified three of the 127 NIBBS seed enzymes. The ones identified by NIBBS were involved in fermentation pathways associated with hydrogen production. Examples of these are: pyruvate synthase (E.C. 1.2.7.1), formate C-acetyltransferase (E.C. 2.3.1.54), and lactate dehydrogenase (E.C. 1.1.1.27).

*Pyruvate Synthase and Formate C-acetyltransferase:* Pyruvate synthase, which is also known as pyruvate: ferredoxin oxidoreductase (PFOR), is the key enzyme for acetyl-CoA formation in many sulfate-reducing, methanogenic, dark fermentative hydrogen-producing bacteria [60]. In strict anaerobic organisms, such as *C. acetobutylicum*, acetyl-CoA pathway is the main route for acetate and hydrogen production. In this pathway, glucose or other sugar molecule is transformed through a series of reactions to generate pyruvate. Pyruvate generated can then be converted to acetyl-CoA by PFOR for synthesis of acetate [4], [35], [61]. In facultative anaerobic bacteria, formate C-acetyltransferase or pyruvate formate lyase (PFL) is utilized to generate formate and acetyl coenzyme A (Acetyl-CoA) [62].

In our study, PFOR was identified by NIBBS as a hydrogen-related enzyme but was missed by Mutual Information. Lack of identification by Mutual Information may be due partly to the fact that two different routes can be utilized by hydrogen producing bacteria. In our experiment, hydrogen producing bacteria were representative of both anaerobic and facultative anaerobic respiration. As such, the presence of multiple phenotypes being expressed may have resulted in inaccuracies by Mutual Information. However, the NIBBS algorithm was able to distinguish the importance of these two enzymes, thus predicting the role of PFOR and PFL in acetate and hydrogen formation.

*Lactate Dehydrogenase:* While identification of enzymes and pathways involved in production of hydrogen is important, one must also understand which pathways may greatly reduce hydrogen yields. One such pathway is the formation of lactate from pyruvate by the enzyme lactate dehydrogenase [32], [61]. In hydrogen production, generation of lactate by bacteria results in decreased hydrogen yields since pyruvate is being directed towards lactate fermentation rather than acetate and butyrate formation [32]. As such, down regulation of lactate dehydrogenase through environmental stressors or genetic manipulation is essential for enhancing bio-hydrogen production.

#### NIBBS seed generation vs. other seed generation algorithms

NIBBS as its first step identifies *seeds* using its *seed generation* algorithm, which are then expanded to phenotype-biased metabolic systems. However, NIBBS can also take as input, seeds obtained using other methods like literature search and statistical tests (T-Test and mutual information [4]). We set up three experiments, dark fermentation organisms vs. light fermentation organisms, dark fermentation organisms vs. hydrogen non-producing organisms and dark fermentation organisms vs. bio-photolysis organisms, to compare the seed sets identified by NIBBS, T-Test, and mutual information (Table S19).

**Figure 4:**
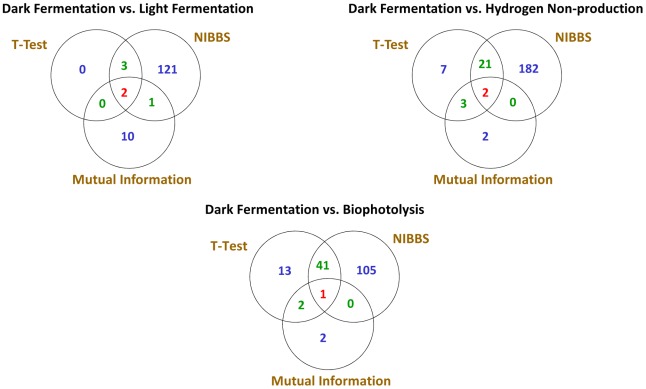
Comparison between NIBBS, T-Test and Mututal Information [**4**].

Mutual information (MI) [4] between the phylogenetic profile of each enzyme and the phenotype profile is considered an indicator of phenotype-bias. An enzyme is considered significantly biased towards a phenotype, if its MI score with the phenotype profile lies above a threshold. The threshold is calculated by shuffling each enzyme vector and calculating its mutual information with the phenotype profile vector. The highest MI value obtained by this process is taken as the threshold. From Figure 4, we can see that mutual information identifies a lot fewer enzymes than NIBBS seed generation algorithm. Additionally, from Table 2, we see that in comparison to NIBBS, mutual information misses all enzymes from the acetate, butyrate and formate pathways that are known to be related to the dark fermentation phenotype. One reason for the low predictive power could be that the filtering mechanism used to identify the significant enzymes is too stringent. Allowing an error margin could improve the predictive power. Another reason could be the fact that mutual information is affected by the size of the vectors used in the calculation, incorporating too many or too few organisms affects the mutual information score.

T-Test is another statistical method that can identify phenotype-related enzymes. Each enzyme's phylogenetic profile is used to calculate the 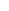-value quantifying its association with the target phenotype. From Figure 4, we can see that T-Test once again identifies a lot fewer enzymes than NIBBS. Additionally, from Table 2, we see that in comparison to NIBBS, T-Test misses some key enzymes from the acetate, butyrate and formate pathways that are known to be related to the dark fermentation phenotype.

### Systematic Validation

In this method we desribe an experiment that evaluates the accuracy of our method using some specialized metabolic pathway information. For this experiment we chose a group of 13 specialized metabolic pathways (Text S1) to act as an artificial phenotype. We then selected around 130 organims that have all these pathways (Text S1). We divided the organisms into two groups, one group was called the “P” and the second group was called the “N.” From the metabolic networks of the organisms belonging to the “N” group, we removed the enzymes that overlap with the chosen metabolic pathways, thus creating an artificial bias. If NIBBS-Search can truely identify phenotype-related subsystems, then it should be able to identify the subsystems related to these metabolic pathways as significant. In fact, we found that all the 13 pathways were significantly present in the discovered subsystems.

### Parameter Evaluation

There are three parameters that the NIBBS algorithm takes as input: (i) the percentage of the positive organisms the resulting subsystem (expanded seed set) should be be present (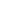), (2) the *maximum bias (maximum* 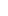 *)*, and (3) the maximum size of the seed set (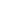). All these parameters have been analyzed using the same artificial dataset created using the 13 specialized metabolic pathways discussed in the *Systematic Validation* section.

The 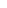 paramater is utilized while performing seed-expansion to control in how many phenotype expressing organisms the resulting expanded seed set should be present. 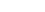 is the most stringent value and would require that the resulting subsystem be present in all of the organisms the seed-set was present in. We utilized this value as default to make sure that only the strongest signals are recorded. However, for this experiment we varied the 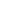 value between 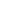 and 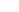 at 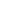 step intervals to analyze the effect. We found that for smaller values of 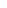, the number of subsystems output are fewer when compared to the larger value of 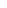. However, for small 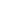 values the subsystem sizes are larger. This effect is due to the fact that more edges get added during the seed-expansion stage because of the lenient (small 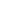) threshold. When we looked at the corresponding phenotype-bias values for the identified subsystems, we found that for a 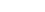, 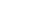 of the systems have phenotype-bias value of less than 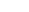, this number steadily decreases until 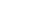 where only 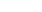 of the subsystems have significant phenotype-bias.

The 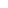 parameter is the maximum seed set size in a NIBBS run. A 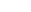 would mean that every candidate seed with a 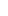 less than the maximum 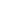 becomes its own seed set and then seed expansion is run on each singleton seed set. We utilized the value 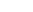 for our experiments. However, we analyzed the effects of 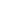 by varying the value between 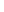 and 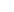 at 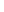 step intervals. We foound that except for 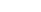, NIBBS identified the 13 specialized metabolic pathways to be signficant for all the other values.

The maximum bias (maximum 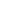) value is chosen to provide an upper bound for the bias value of the enumerated subsystems. We varied the maximum bias value between 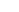 and 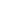 in 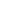 step intervals. Fro example, setting the maximum bias value as 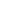 will enumerate all the subsystems with final bias value of 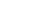. We found that the number of subsystems produced for a maximum bias value 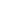 is greater than or equal to the number of subsystems produced for maximum bias value of 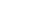. The analysis and data related to this section are available in Text S2.

### Runtime Performance

In order to display the dramatic improvement in the runtime of the NIBBS-Search algorithm over exact algorithms, such as MBS-Enum , 98 organism-specific networks are constructed using the global metabolic reference map from the KEGG database [15]–[17], which contains 1,348 vertices and 1,476 edges: 50 metabolic networks from aerobic organisms and 48 metabolic networks from anaerobic ones.

The MULE algorithm of Koyutürk *et al.* [63] is used to enumerate maximal frequent subgraphs for all support count thresholds between 1 and the number of positive instances required by MBS-Enum . MULE is selected because both MBS-Enum and NIBBS-Search leverage its network instance model. Such a model allows MULE to enumerate maximal frequent subgraphs by enumerating maximal frequent edge sets, which makes it one of the most efficient methods for enumerating maximal frequent subgraphs [63]. The MBS-Enum is not a wrapper around the MULE algorithm.

**Figure 5:**
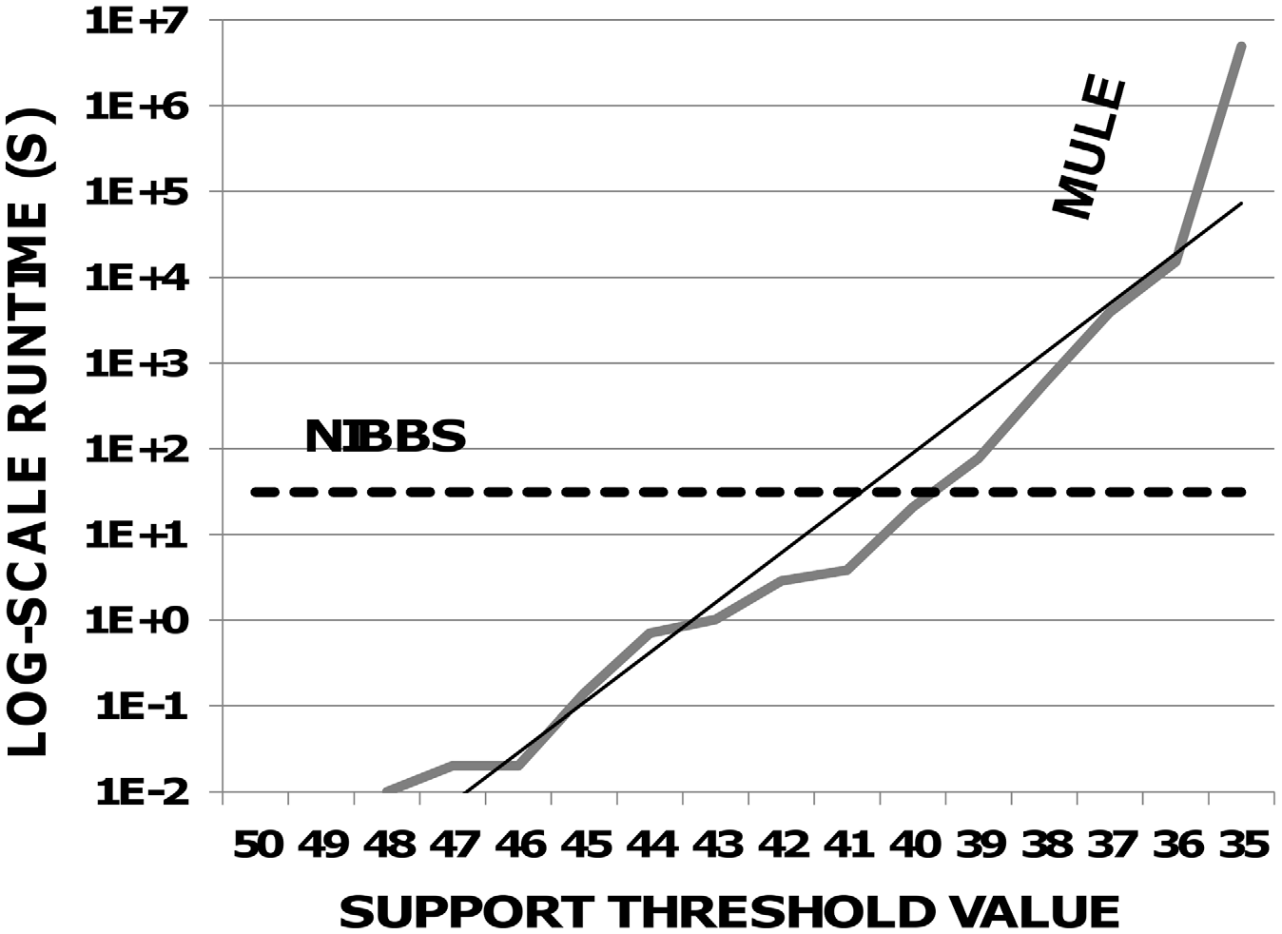
MULE vs. NIBBS runtime comparison. Runtimes (y-axis), with trendline, of the MULE algorithm for the various support count thresholds (x-axis) used by the MBS-Enum algorithm. Total runtime required by the NIBBS-Search algorithm drawn as horizontal dotted line.

Even using the efficient MULE algorithm, the runtime of MBS-Enum is intractable for the large-scale networks in this experiment. Figure 5 (Table S20) depicts the MULE runtime for the various thresholds used by MBS-Enum . This runtime grows exponentially, eventually reaching 57 days to enumerate the maximal frequent subgraphs given a support count threshold of 35. In contrast, the *total* time required by the NIBBS-Search to approximate the set of maximally-biased subgraphs is 31 seconds (the dotted line).

### Approximation Accuracy

The results in this section describe the typical correspondence between the set of subgraphs output by the NIBBS-Search and the complete set of maximally-biased subgraphs produced by MBS-Enum (Table S21). To cope with computational intractability of MBS-Enum, only small-size network maps are considered. Specifically, the 33 experiments correspond to the 33 metabolic pathway maps from KEGG that satisfy the two requirements: (1) all of their maximally-biased subgraphs can be enumerated by MBS-Enum within 24 hours; (2) a completely random subgraph can be generated by a randomization algorithm at a rate of at least one per second. For each of these 33 network maps, a set of 87 network instances are created. These 87 network instances are divided between 33 positive instances for aerobic organisms and 54 negative instances for anaerobic organisms. Each experiment is labeled with the KEGG pathway identifier (mapXXXXX) of the network map used to create the network instances.

An approximation score 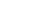 is used to measure the degree to which a set of NIBBS-Search 's subgraphs 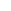 approximates a set of all maximally-biased subgraphs 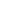. The approximation score is calculated by first computing the value 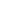 for each maximally-biased subgraph in 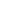. The value 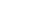 is equal to the maximum Jaccard index (Equation 1) between a maximally-biased subgraph 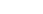 and any subgraph 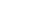 (Equation 2). The appoximation score 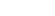 is then calculated as the normalized Euclidean distance between the scores 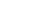 computed for the set of NIBBS-Search 's subgraphs and the optimal 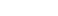.
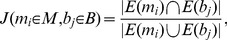
(1)

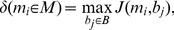
(2)

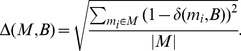
(3)


**Figure 6:**
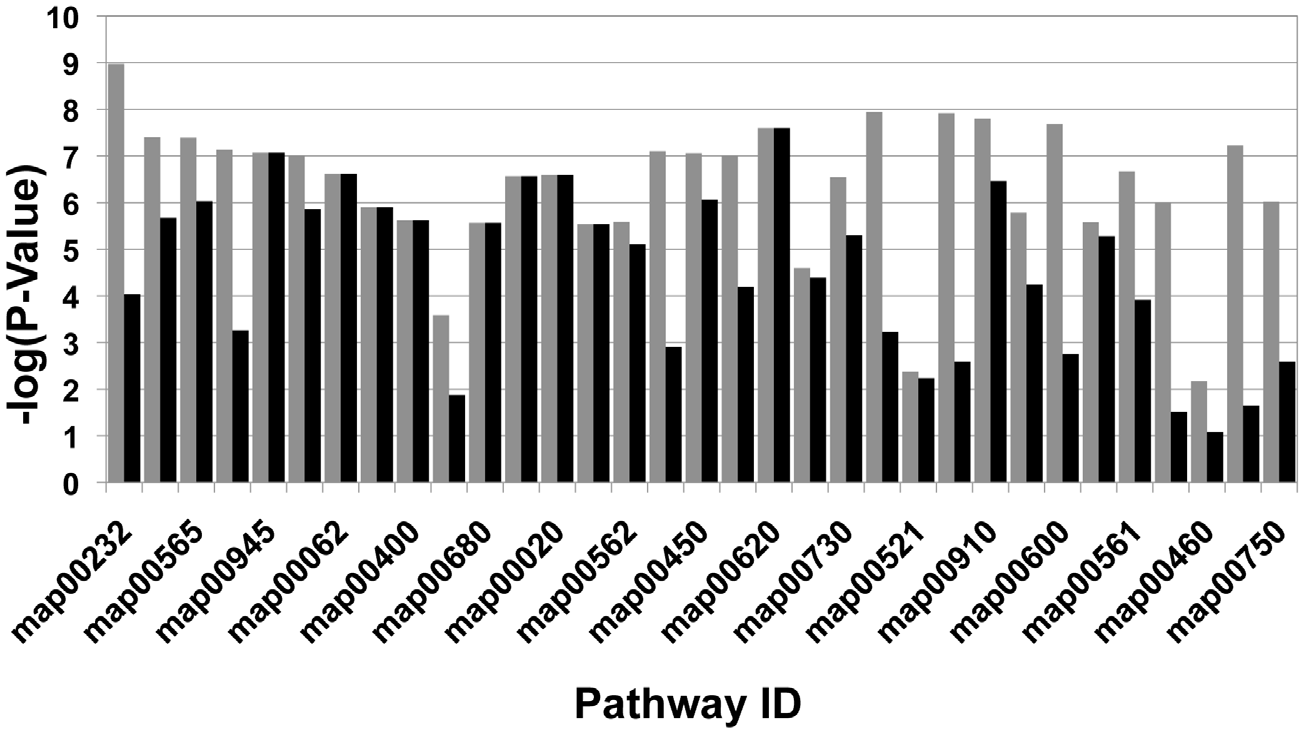
Approximation Accuracy: The negative log of the statistical significance of the approximation scores has been plotted. The 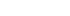 for 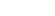. Gray: 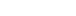; black: 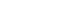.

Two empirical 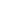-values are calculated to determine the statistical significance of the approximation scores. Both 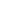-values are calculated as the empirically-determined probability that a set of randomly generated subgraphs 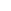 would generate a value 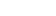 that is less than or equal to the value of 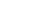. Each randomly generated set of subgraphs 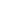 contains the same number of random subgraphs as the set 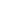. The random subgraphs used to calculate the 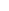-value, 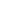, are randomly selected from the set of connected subgraphs in the network map associated with the experiment. For the 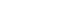, the random subgraph 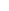 of the set 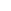 is required to be of the same size as the NIBBS-Search's subgraph 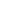 from the set 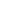. By ensuring that the random subgraphs are of the same size as the NIBBS-Search's subgraphs, the calculation of 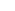 addresses some of the noise that might arise in the 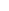-value when the random subgraphs are of a different scale than the NIBBS-Search 's subgraphs. The negative-logs of the empirical values of 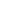 and 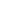 are shown for each of the 33 experiments in Figure 6.

As can be seen in Figure 6, 100 percent of the experiments had a 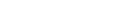. In addition, 88% of the experiments had a 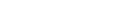. These results give strong support to the claim that NIBBS-Search identifies subgraphs that are typically close approximations of the set of maximally-biased subgraphs. Thus, if maximally-biased subgraphs are a good model of phenotype-related metabolic systems, NIBBS-Search should be able to identify them as models of phenotype-related metabolic systems.

## Discussion

In summary, the NIBBS search algorithm was able to identify phenotype-related metabolic pathways and sub-networks across sets of phenotype-expressing microorganisms. Specifically, through co-development and application of the NIBBS algorithm, both pathways specific to and those related to dark fermentative, hydrogen production and acid-tolerance were presented. From those identified pathways, scientists are able to gain insight into the potential role some pathways, such as fatty acid metabolism, have on metabolic shifts between hydrogen production and solvent formation.

In addition, through comparison of multiple phenotypes deemed important for hydrogen production in wastewater, pathways responsible for expression of more than one phenotype were identified. Specifically, pathways for purine metabolism and the pathways for proline and arginine metabolism were predicted as related to dark fermentative hydrogen production and acid-tolerance. Due the continued presence of these two pathways, engineers and scientists can experimentally test the role of the pathways as survival mechanisms for acid response and hydrogen production. Identification of these shared pathways for the two phenotypes is due to the ability of the multiple organisms to express multiple phenotypes. For example, *Clostridium acetobutylicum* ATCC 824 and *Clostridium perfringens* ATCC 13124 are both dark fermenting organisms but they also share other common phenotypes like anaerobicity and tolerance to acid. These phenotypes if analyzed as a group, may provide us more information about the phenotype systems in these two organisms than looking at each phenotype one by one.

### Implications for Microbial Metabolic Engineering

Application of the NIBBS-Search algorithm to the hydrogen producing and acid-tolerant phenotypes resulted in the prediction of potentially important enzymes, metabolic pathways, and key regulators involved in maintaining or enhancing the production of hydrogen in individual microorganisms. Such predictions include pathways, such as fatty acid biosynthesis, which may help hydrogen producers respond to pH changes both internally and externally. The response to both the formation and uptake of fatty acids present in the surrounding environment suggests that fatty acid biosynthesis could potentially act as a key regulator in metabolic shifts in microorganisms, such as *C. acetobuylicum*. Other examples provided by NIBBS included the presence or absence of acid tolerant systems and enzymes within specific *Clostridium* species. In this study, results indicate that *C. perfringens* contains potentially important enzymes involved in the acid-tolerant ADI pathway. The identified enzymes may then suggest clues necessary for development of gene expression and molecular validation studies.

### Identification of Potential Metabolic Pathway Cross-talks

In addition to identifying conserved metabolic pathways, results from the NIBBS algorithm suggest that this method can potentially identify metabolites common to different metabolic pathways. One example of such a metabolite is acetyl-CoA. Acetyl-CoA is generated from pyruvate during glycolysis and can be utilized by differing pathways, including the aerobic TCA cycle and anaerobic formate hydrogen lyase pathway. In the aerobic TCA pathway, the enzyme, pyruvate dehydrogenase, catalyzes the decarboxylation of pyruvate to 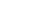 (g) and acetyl-CoA. Acetyl-CoA generated using this process can then be incorporated into the TCA cycle to produce important biosynthetic precursors for other metabolic pathways and energy for microorganisms [34], [64]. In the anaerobic pathway, pyruvate formate lyase is used to convert pyruvate into acetyl-CoA and formate. Formate produced can then be oxidized by formate hydrogen lyase (FHL) to form 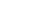 (g) and 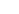 (g). In the hydrogen studies, the NIBBS algorithm predicts the presence of both pyruvate formate lyase (E.C. 1.1.99.3) and pyruvate dehydrogenase (E.C. 1.2.4.1) when dark fermentative hydrogen producing organisms are compared to hydrogen non-producing organisms. The presence of both pathways may be due to the fact that some dark fermentative microorganisms are capable of utilizing both pathways and the degree to which they utilize each pathway may be dependent on the “cross-talk” between both pathways. However, depending on environmental conditions, the bacteria are grown under, the organism may be more prone to express one phenotype over the other. To understand the role of these pathways, further experimental analysis is required.

Identification of common metabolites and potential cross-talk between metabolic pathways is a key step towards understanding metabolic processes, networks, and regulation of phenotype expression in organisms, such as hydrogen producing organisms. While numerous genetic and experimental studies have been conducted to understand the metabolic processes involved in hydrogen production, there is still little understanding of the cross-talk between key hydrogen producing pathways. To help close this gap, biologist could potentially use the NIBBS algorithm to complement hypothesis-driven studies. One way would be to identify phenotype related-pathways, such as the two pathways for acetyl-CoA production, and then conduct molecular studies to review these pathways in organisms shown positive for both pathways.

### Multiple Phenotypes vs. Single Phenotype

The idea of identifying phenotype-related systems has always been of interest to scientists for many years now and almost all existing methodologies look at phenotypes one at a time. The only method that looks at more than one phenotype, to the best of our knowledge, is the one presented by Liu *et al*, [65] but even here, the authors primarily look at one phenotype at a time and then use the Pfam-phenotype relationship discovered to identify groups of related phenotypes. Liu *et al* [65], however, also do not analyze the effects of multiple phenotypes simultaneously. *Clostridium acetobutylicum* and *Clostridium perfringens* have both dark fermenting organisms, but they also share other common phenotypes like anaerobicity and tolerance to acid. These phenotypes, if analyzed as a group, may provide us more information about the phenotype systems in these two organisms than if they were looked at individually. A future improvement could be for NIBBS to analyze multiple phenotypes together.

### Phylogenetic Diversity

In any comparative genomics, there is always the question whether the identified modules are truely related to the phenotype or they were identified because the organisms are phylogenetically close to each other. Incorporating a method to identify not only phenotypically-biased organisms but also subsyetems present across a phylogenetically diverse group might be one future improvement. This probably can be done by creating a metric that will use the pair-wise phylogetic distances among all the organisms the subsystem is present in. A subsystem present across a phylogenetically diverse group should be scored higher than one that is present across a phylogenetically similar group of organisms.

The quality of NIBBS results is also dependent on the underlying data. We discussed one issue in the previous paragraph about phylogenetic diversity. Another issue is the fact that the quality of the results is also dependent on high-quality enzyme-reaction associations. However, databases like KEGG, MetaCyc, and BioCyc provide fairly standard data that can be utilized for such an analysis.

## Methods

This approach aims to comparatively search the metabolic network of multiple phenotype-expressing and phenotype-non-expressing organisms for systems that tend to be present in the former but not present in the latter. The underlying hypothesis is that a phenotype-related metabolic system is more likely to be evolutionarily conserved across phenotype-expressing organisms, thus it is *phenotype-biased*. This section explains the NIBBS methodlogy. Additional details can be found in Matthew C. Schmidt's doctoral dissertation [66].

### Network Model

The proposed approach requires a metabolic network model that enables:

1. The definition of organism-specific networks for hundreds or thousands of organisms.
2. The quick determination if a metabolic system is present in an organism-specific network.
3. The definition of the set of metabolic systems that could possibly exist in an organism.

To satisfy these requirements, we adapt the method of modeling organism-specific networks introduced by Koyutürk *et al.* [67]. Derived from the KEGG database [15]–[17], non-organism-specific, yet biochemically feasible, metabolic networks, or *reference* maps, are modeled as networks whose vertices represent chemical compounds, or metabolites, and whose edges represent reactions that convert metabolites to products. The reaction set corresponds to the set of known reactions that can perform such a conversion. Each reaction is associated with an Enzyme Commission (EC) number [68] that is also associated with enzymes that can catalyze the reaction.

While metabolic reference maps capture every known, biochemically feasible metabolic process, *organism-specific* networks describe the metabolic network that exists in a given organism. Specifically, every edge in such a network is associated with an EC number of the enzyme that is known or predicted to be present in the organism. We obtain the *organism-specific* networks from the *reference* maps by retaining only those reactions that are catalyzed by an enzyme present in the organism, i.e, by retaining only those edges whose edge labels represent enzymes present in the organism.

A subgraph is said to exist in an organism-specific network, if the edge lables, i.e., the enzymes are present in the organism. Thus, we do not solve any subgraph isomorphism problem. In addition, with this model, the set of all possible metabolic systems can be defined as the set of subgraphs of the reference map. Moreover, only connected subgraphs need to be considered, because metabolic systems are defined as a series of metabolic reactions, where the product metabolites of one reaction are used as the substrate metabolites of the next reaction.

### Bias Metric

The introduced 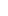-value of a metabolic system measures the degree of a system's phenotype-bias. It is based on the hypothesis that the systems with the greatest degree of bias (i.e., smaller 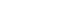) will be the systems that are most likely to be phenotype-related. Thus, the search for phenotype-related metabolic systems will aim to minimize the 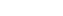.

To calculate the 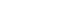 for a given system, the organism-specific networks are divided into two sets: those for phenotype-expressing organisms, or a positive set, and those for phenotype-non-expressing organisms, or a negative set.

*Given the number of organism-specific networks (* 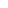 *), the number of positive networks (* 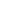 *), the number of networks that the system exists in (* 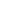 *), and the number of positive networks the system exists in (* 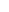 *), the phenotype-bias metric* 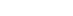 *is defined according to the cumulative hypergeometic probability distribution:*

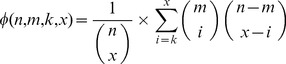
(4)


Because 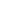, 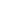, 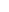, and 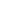 can be determined given the system subgraph 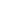 and the set of positive 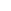 and negative 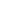 networks, the 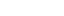 notation will also be used to describe the phenotype-bias metric.

### Maximally-Biased Subgraphs

In order to predict phenotype-related metabolic systems, this approach searches the set of organism-specific networks for maximally-biased subgraphs.

*A maximally-biased subgraph is a subgraph that satisfies the following two criteria:*

1. *It has no subgraph whose* 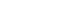 *is less than its own's.*
2. *It has no supergraph whose* 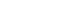 *is less than or equal to its own's.*

The first criterion comes from the assumption that the entire phenotype-related system is at least as biased as its smaller part. The second criteria is the one that makes the reported subgraphs maximal. According to the second criteria, only allowing those subgraphs that have no larger subgraph with equal or smaller bias are reported.

### Algorithm

This section presents the Maximally-Biased Subgraph Enumeration (MBS-Enum) and the Network Instance Based Biased Subgraph Search ( NIBBS-Search ) algorithms that respectively enumerate the exact and the approximate set of maximally-biased subgraphs as models of phenotype-related metabolic systems. While being exact, MBS-Enum becomes computationally intractable for genome-scale networks. In contrast, NIBBS-Search is a fast heuristic, suitable for hundreds of genome-scale networks; yet, it produces a statistically close approximation of the full set when empirically tested against MBS-Enum results generated for small-scale networks.

#### The MBS-Enum algorithm

Before presenting the MBS-Enum algorithm for exact enumeration of all maximally-biased subgraphs, we first define some graph-theoretical terms. A subgraph 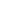 exists in a network if it contains a subgraph that is isomorphic to 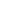. The number of networks that a subgraph is present in is called the *support count* of the subgraph. Given a set of networks 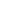 and a subgraph 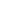, the support count of 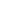 is labeled as 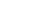. A *frequent subgraph* is any subgraph whose support count is greater than or equal to a given threshold. A maximal frequent subgraph is a frequent subgraph that is not a subgraph of any larger frequent subgraph.

The Maximally-Biased Subgraph Enumeration algorithm (MBS-Enum) enumerates all maximally-biased subgraphs for a set of network instances 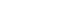. MBS-Enum first enumerates all maximal frequent subgraphs for the set of positive networks 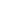 and every threshold 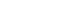. It then filters this set by removing non-maximally-biased subgraphs.

MBS-Enum enumerates all maximally-biased subgraphs if and only if every maximally-biased subgraph is also a maximal frequent subgraph for some threshold 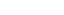. To prove this theorem, note that the following two properties of the bias metric are true:

1. If 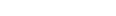 and 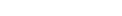, then 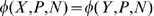;
2. If 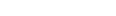 and 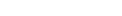, then 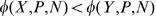,

where 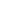 and 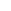 are subgraphs, 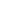 and 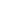 are the respective positive and negative sets, and 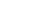 is the support count of 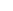 in 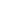.

**Theorem:** *A maximally-biased subgraph* 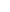 *for given positive* 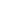 *and negative* 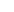 *sets of networks is a maximal frequent subgraph for the threshold*

*Proof:* Let 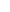 be a maximally-biased subgraph. Assume that 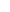 is not a maximal frequent subgraph for the set 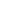 and threshold 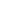 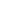 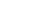. Then there must exist a subgraph 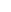, such that 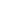 is a frequent subgraph for 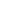 and 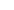 and 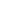 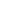 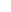. Since 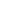 is a supergraph of 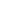 and 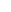 is a frequent subgraph for 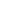 and 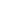, 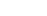 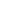 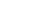. The fact that 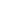 is a supergraph of 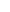 means that 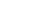 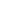 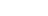. Due to the properties of the bias metric listed above, 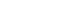 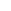 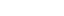. This means that 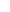 cannot be a maximally-biased subgraph, because 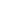 is a supergraph and has a 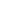-value that is less than or equal to that of 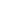, which is a violation of the second property of maximally-biased subgraphs. Thus, the original assumption must be incorrect, and 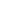 must be a maximal frequent subgraph for 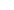.

#### The NIBBS-Search algorithm

A general overview of the NIBBS-Search algorithm is given in Algorithm 1 in Text S3. It is a two-step process that first identifies small seed sets of edges and then expands those sets into the maximally-biased subgraphs.

#### Seed set generation

**Figure 7:**
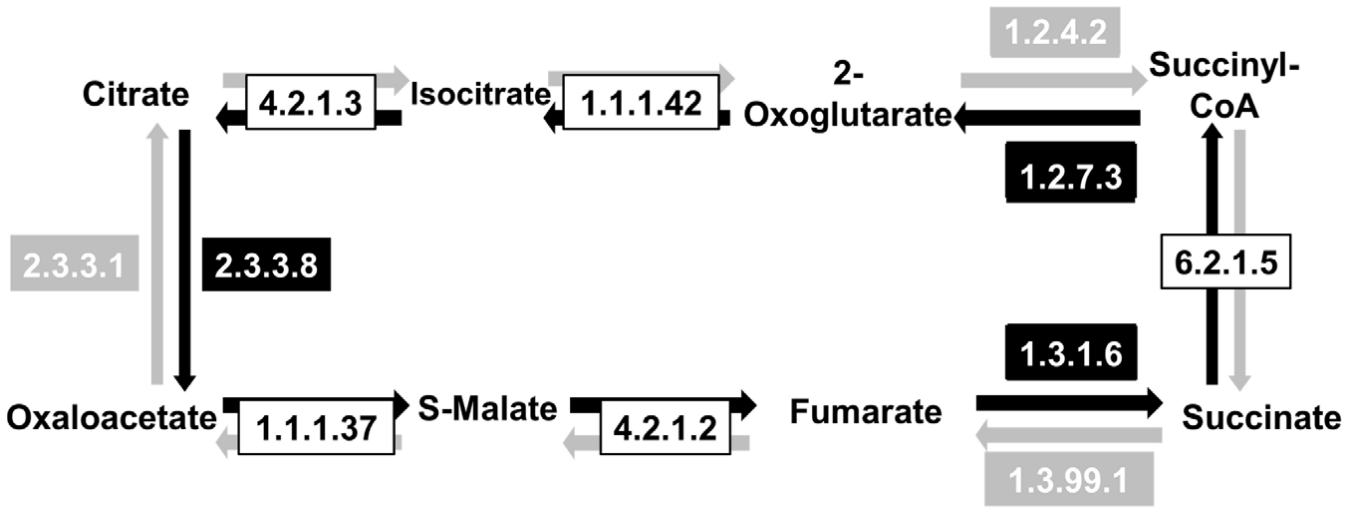
TCA and rTCA metabolic pathways. TCA cycle: gray arrows; rTCA: black arrows; EC numbers: white boxes; Pathway specific EC numbers: TCA-specific (gray), rTCA-specific (black).

Informally, *seed sets* correspond to significant subsets of edges from the network map; they differentiate between common subgraphs that model phenotype-related systems and those that model phenotype un-related systems, and they improve the NIBBS-Search efficiency by determining the subset of organisms that are predicted to contain the entire phenotype-related system. The motivation behind seed set generation stems from the following observation. The phylogenetic profile of a phenotype-related metabolic system, such as the tricarboxylic acid (TCA) and reverse TCA (rTCA) cycle is often the same as the phylogenetic profile of a small subset of its constituent enzymes (Figure 7 and Table S16). In other words, this subset defines the set of target organisms that contain the entire system, and thus reduces the set of network instances that need to be aligned during the expansion process. In addition, it provides hints to the algorithm that among the possibly many common subgraphs that are found when the instances are aligned, only those that contain the seed set should be predicted to represent phenotype-related systems.

The procedure implemented in the NIBBS-Search algorithm for growing seed sets is given in Algorithm 2 in Text S3 It begins by sorting the set of edges in the network map by their 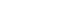 (Line 1) Then the edge with the least 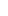-value is used to create a seed set containing only that edge (Line 3) To avoid redundant seed sets, that edge is marked, so it cannot be added to any other seed set (Lines 5 and 10) The *GenerateSeedCandidates* identifies a set of *candidate edges* (Line 6), which are the edges whose addition to the seed set decreases its 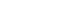. Only unmarked edges are considered as possible candidate edges. A candidate edge that produces the greatest decrease to the seed set's 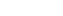 is termed as a “best” candidate. The algorithm follows a greedy approach by adding these “best” edges to the seed set (Line 8). After an edge is added to the seed set, the set of candidate edges is updated (Line 11). This process continues until the 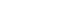 of the seed set cannot be decreased by adding any candidate edge, or until the seed set reaches a user-defined maximum size (Line 7). The seed set is then added to the set of seed sets, and a new seed set is generated from the unmarked edge that has the least 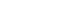. This process continues until every edge in the network map is part of a seed set.

Two methods of selecting the candidate edges are defined. The first ensures that the seed set forms a connected subgraph. The second does not require that the seed set be connected but ensures that the seed set be part of a connected subgraph after the expansion process. The first method is achieved by only considering edges that are adjacent with one of the edges currently in the seed set. The second method considers any edge in the network map as a candidate edge as long as the two edges are connected after the expansion process. To ensure that the two edges are connected, the method determines if there exists a path between the edge and one of the edges in the seed set that is present in every positive network instance that the new seed set would be present in.

The user chooses a threshold 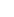 such that only seed sets whose 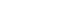 is less than 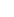 will be expanded into full subgraphs. This allows the user to reduce the number of insignificant subgraphs that are output by the algorithm. Due to the method by which the seed sets are constructed, every edge in the network map will be part of at least one seed set.

#### Seed set expansion

The seed set of edges is unlikely to represent the entire phenotype-related metabolic system. Seed sets are typically small, containing between one and five edges and, depending on the method used to construct them, may form a disconnected subgraph. A metabolic system is likely to form a connected subgraph in a metabolic network containing many more edges [69]. In order to predict the entire set of enzymes belonging to the metabolic system, the NIBBS-Search algorithm expands the seed sets. To ensure that the expansion edges belong to the same metabolic system as the seed edges, the expansion process requires that the expansion edges be present in most if not all of the metabolic networks of phenotype-expressing organisms that also contain the seed edges. The addition of expansion edges to a seed set to form the subgraphs output by the algorithm is called the seed expansion process. During the process, an expansion edge is selected from a set of candidate edges. These candidate edges are determined by two criteria checked in the *GenerateExpansionCandidates* function (Line 1 and Line 5):

1. They are adjacent to a seed edge or an expansion edge is already in the edge set.
2. If added to the current edge set, the resulting edge set will be present in at least 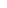 percentage of the positive network instances that the seed set was present in.

The first criterion ensures that the final edge set will form a connected subgraph. The second criterion allows for noise in the data, while requiring that the final edge set still be present in most if not all of the same positive network instances as the seed set. The algorithm for expanding the seed set to form the final edge sets is given in Algorithm 3 in Text S3. Expansion edges are selected from the set of candidate edges, added to the current edge set, and the set of candidate edges is updated until no candidate edges can be found. The resulting edge set is then output.

The order in which candidate edges are added to the edge set will determine the make-up of the output edge set unless 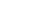. The expansion process determines which candidate edge to add to the edge set by first considering the number of positive network instances that the resulting edge set would be present in. It selects the candidate edge that would maximize the number of positive instances the resulting edge set is present in. However, multiple candidate edges may exist that would result in edge sets present in the same number of phenotype-expressing organisms. In this case, the expansion process selects from this set the candidate edge that would produce the greatest decrease in the 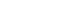 of the edge set. If more than one of these candidates produce the same decrease in the 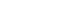, then a candidate edge is selected at random from these remaining candidates and added to the edge set.

Every NIBBS-Search run uses the maximum seed set size 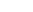, the maximum 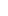-value for expansion 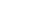, and the subgraph expansion parameter 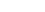. Running NIBBS-Search with those parameters identifies subgraphs that most closely approximate maximally-biased subgraphs.

### Identification of Enriched Pathways

The hypergeometric test is utilized to identify the pathways enriched by the metabolic subsystems identified by NIBBS for the hydrogen production, dark fermentation, and acid tolerance phenotypes. The enriched pathways are identified for *Clostridium acetobutylicum* as follows. The edges in all the subsystems are combined into one list 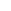 and the duplicates are removed. For each metabolic pathway 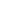, the edges in the KEGG *reference* pathway map form the population 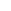. The edges in the *organism-specific* pathway map of 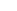 become successes 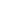 in the population. The edges in 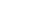 become the sample 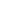 and 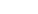 are the successes 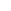 in the sample.

## Supporting Information

Table S1 **Organisms used in the experiments.** This file consists of information regarding the organisms utilized in the various experiments.
(XLS)

Table S2 **NIBBS-Search results for the hydrogen production phenotype.** This file consists of NIBBS-Search results for using hydrogren production as target phenotype.
(TXT)

Table S3 **NIBBS-Search results for the dark fermentation, hydrogen production phenotype versus bio-photolysis, hydrogen production phenotype.** This file consists for NIBBS-Search results when using dark fermentation as target phenotype and bio-photolysis organisms set was used as the “negative set.”
(TXT)

Table S4 **NIBBS-Search results for the dark fermentation, hydrogen production phenotype versus light fermentation, hydrogen production phenotype.** This file consists for NIBBS-Search results when using dark fermentation as target phenotype and light fermentation organisms set was used as the “negative set.”
(TXT)

Table S5 **NIBBS-Search results for the dark fermentation, hydrogen production phenotype versus hydrogen non-production organisms.** This file consists for NIBBS-Search results when using dark fermentation as target phenotype and hydrogen non-producing organisms set was used as the “negative set.”
(TXT)

Table S6 **NIBBS-Search results for the light fermentation, hydrogen production phenotype versus bio-photolysis, hydrogen production phenotype.** This file consists for NIBBS-Search results when using light fermentation as target phenotype and bio-photolysis organisms set was used as the “negative set.”
(TXT)

Table S7 **NIBBS-Search results for the light fermentation, hydrogen production phenotype versus dark fermentation, hydrogen production phenotype.** This file consists for NIBBS-Search results when using light fermentation as target phenotype and dark fermentation organisms set was used as the “negative set.”
(TXT)

Table S8 **NIBBS-Search results for the light fermentation, hydrogen production phenotype versus hydrogen non-production organisms.** This file consists for NIBBS-Search results when using light fermentation as target phenotype and hydrogen non-producing organisms set was used as the “negative set.”
(TXT)

Table S9 **NIBBS-Search results for the bio-photolysis, hydrogen production phenotype versus dark-fermentation, hydrogen production phenotype.** This file consists for NIBBS-Search results when using bio-photolysis as target phenotype and dark fermentation organisms set was used as the “negative set.”
(TXT)

Table S10 **NIBBS-Search results for the bio-photolysis, hydrogen production phenotype versus light fermentation, hydrogen production phenotype.** This file consists for NIBBS-Search results when using bio-photolysis as target phenotype and light fermentation organisms set was used as the “negative set.”
(TXT)

Table S11 **NIBBS-Search results for the bio-photolysis, hydrogen production phenotype versus hydrogen non-production organisms.** This file consists for NIBBS-Search results when using bio-photolysis as target phenotype and hydrogen non-producing organisms set was used as the “negative set.”
(TXT)

Table S12 **Metabolic pathways of** ***Clostridium acetobutylicum*** **enriched by the subsystems identified by NIBBS-Search for the dark fermentation, hydrogen production phenotype.** This file has the results described in section *Pathways Related to Dark Fermentative Hydrogen Production*.
(XLS)

Table S13 **NIBBS-Search results for the acid-tolerent phenotype.** This file has the NIBBS-Search results for utlizing acid-tolerence as target phenotype.
(TXT)

Table S14 **Enzymes related to acid-tolerent phenotype.** This file consists of a comparison between enzymes in acid-tolerant organisms and alkaliphilic (non-acid-tolerant) organisms, in acid-tolerant experiments. Each row represents enzymes identified by NIBBS-Search and their corresponding pathways they are present in. The results are discussed in section *Acid-tolerant Enzymes*.
(PDF)

Table S15 **Metabolic pathways of** ***Clostridium acetobutylicum*** **enriched by the subsystems identified by NIBBS-Search for the acid-tolerent phenotype.** This file consists of results described in section *Acid-tolerant Pathways*.
(XLS)

Table S16 **Enzymes related to TCA/rTCA expression.** This file consists of a comparison, presence (+) or absence (−) of enzymes across the set of organisms used in the TCA and rTCA experiments.
(PDF)

Table S17 **NIBBS-Search results for TCA versus rTCA expression.** This file consists for NIBBS-Search results when using TCA expression as target phenotype and rTCA expressing organisms set was used as the “negative set.”
(TXT)

Table S18 **NIBBS-Search results for rTCA versus TCA expression.** This file consists for NIBBS-Search results when using rTCA expression as target phenotype and TCA expressing organisms set was used as the “negative set.”
(TXT)

Table S19 **Comparison of NIBBS-Search seed generation algorithm to other seed generation algorithms for the dark fermentation, hydrogen production phenotype.** This file consists a comparative analysis between the enzymes identified for dark fermenatation, hydrogen production phenotype by NIBBS to the enzymes identified for the same target phenotype by T-Test and Mutual information.
(XLS)

Table S20 **Runtime comparisons.** This file consists of the results of runtime comparison between NIBBS-Search and other algorithms.
(XLSX)

Table S21 **Correspondence between outputs of NIBBS-Search and MBS-Enum.** This file consists of the results of the experiment evaluating the corresspondence of subgraphs output by NIBBS to the set of complete set of maximally-biased subgraphs output by MBS-Enum.
(XLSX)

Text S1 **Systematic validation.** This file contains the result of the experiments relating to the accuracy evalutaion of NIBBS-Search.
(BZ2)

Text S2 **Parameter evaluation.** This file contains the results of the experiments performed to analyze the effects of input parameters on the results.
(BZ2)

Text S3 **NIBBS-Search Algorithm Pseudocode.** This file contains more details on the NIBBS-Search algorithm.
(PDF)

## References

[1] Alvira P, Tomás-Pejó E, Ballesteros M, Negro M (2010). Pretreatment technologies for an efficient bioethanol production process based on enzymatic hydrolysis: A review.. Bioresour Technol.

[2] Galbe M, Zacchi G (2002). A review of the production of ethanol from softwood.. Appl Microbiol Biotechnol.

[3] Santos C, Stephanopoulos G (2008). Combinatorial engineering of microbes for optimizing cellular phenotype.. Curr Opin Chem Biol.

[4] Slonim N, Elemento O, Tavazoie S (2006). Ab initio genotype-phenotype association reveals intrinsic modularity in genetic networks.. Mol Syst Biol.

[5] Jim K, Parmar K, Singh M, Tavazoie S (2004). A Cross-genomic approach for systematic mapping of phenotypic traits to genes.. Genome Res.

[6] Benfey P, Mitchell-Olds (2008). From genotype to phenotype: Systems biology meets natural variation.. Science.

[7] Bailey J, Sburlati A, Hatzimanikatis A, Lee K, Renner W (2002). Inverse metabolic engineering: A strategy for directed genetic engineering of useful phenotypes.. Biotechnol Bioeng.

[8] Hartwell L, Hopfield J, Leibler S, Murray A (1999). From molecular to modular cell biology.. Nature.

[9] Kelley B, Sharan R, Karp R, Sittler T, Root D (2003). Conserved pathways within bacteria and yeast as revealed by global protein network alignment.. Proc Natl Acad Sci U S A.

[10] Flannick J, Novak A, Srinivasan B, McAdams H, Batzoglou S (2006). Graemlin: General and robust alignment of multiple large interaction networks.. Genome Res.

[11] Tian W, Samatova N (2009). Pairwise alignment of interaction networks by fast identification of maximal conserved patterns..

[12] Chen W, Schmidt M, Tian W, Samatova N (2009). A fast, accurate algorithm for identifying functional modules through pairwise local alignment of protein interaction networks..

[13] Chen W, Rocha A, Hendrix W, Schmidt M, Samatova N, Fan W, Hsu W, Webb G, Liu B, Zhang C (2010). The multiple alignment algorithm for metabolic pathways without abstraction.. ICDM Workshops.

[14] Levesque M, Shasha D, Kim W, Surette M, Benfey P (2003). Trait-to-gene: A computational method for predicting the function of uncharacterized genes.. Curr Biol.

[15] Kanehisa M, Goto S, Furumichi M, Tanabe M, Hirakawa M (2010). KEGG for representation and analysis of molecular networks involving diseases and drugs.. Nucleic Acids Res.

[16] Kanehisa M, Goto S, Hattori M, Aoki-Kinoshita K, Itoh M (2006). From genomics to chemical genomics: New developments in KEGG.. Nucleic Acids Res.

[17] Kanehisa M, Goto S (2000). KEGG: Kyoto encyclopedia of genes and genomes.. Nucleic Acids Res.

[18] Khanal S, Khanals S (2008). Biohydrogen production: Fundamentals, challenges, and operation strategies for enhanced yield.. Anaerobic biotechnology for bioenergy production: Principles and applications.

[19] Dabrock B, Bahl H, Gottschalk G (1992). Parameters affecting solvent production by Clostridium pasteurianum.. Appl Environ Microbiol.

[20] Nath K, Das D (2004). Improvement of fermentative hydrogen production: Various approaches.. Appl Microbiol Biotechnol.

[21] Kapdan I, Kargi F (2006). Bio-hydrogen production from waste materials.. Enzyme Microb Technol.

[22] Land Peccia BrentnerJ, Zimmerman J (2010). Challenges in developing biohydrogen as a sustainable energy source: Implications for a research agenda.. Environ Sci Technol.

[23] Li C, Fang H (2007). Fermentative hydrogen production from wastewater and solid wastes by mixed cultures.. Crit Rev Environ Sci Technol.

[24] Hallenbeck P, Benemann J (2002). Biological hydrogen production; fundamentals and limiting processes.. Int J Hydrogen Energy.

[25] Nandi R, Sengupta S (1998). Microbial production of hydrogen: An overview.. Crit Rev Microbiol.

[26] Rand Fang LiH (2009). Hetertrophic photoferementative hydrogen production.. Crit Rev Environ Sci Technol.

[27] Yu J, Takahashi P, Mendez-Vilas A (2007). Biophotolysis-based hydrogen production by cyanobacteria and green microalgae.. Communicating Current Research and Educational Topics and Trends in Applied Microbiology. volume 1.

[28] Khanal S, Khanal S (2008). Bioenergy generation from residues of biofuel industries.. Anaerobic biotechnology for bioenergy production: Principles and applications.

[29] Claassen P, van Lier J, Lopez Contreras A, van Niel E, Sijtsma L (1999). Utilisation of biomass for the supply of energy carriers.. Appl Microbiol Biotechnol.

[30] Rey F, Heiniger E, Harwood C (2007). Redirection of metabolism for biological hydrogen production.. Appl Environ Microbiol.

[31] Miyake J (1998). Biohydrogen.

[32] Hallenbeck P, Ghosh D (2010). Improvements in fermentative biological hydrogen production through metabolic engineering.. J Environ Manage.

[33] Jones P (2008). Improving fermentative biomass-derived H2 production by engineering microbial metabolism.. Int J Hydrogen Energy.

[34] White D (2007). The physiology and biochemistry of prokaryotes.

[35] Lee J, Yun H, Feist A, Palsson B, Lee S (2008). Genome-scale reconstruction and in silico analysis of the ***Clostridium acetobutylicum*** ATCC 824 metabolic network.. Appl Microbiol Biotechnol.

[36] Huang L, Forsberg C, Gibbins L (1986). Inuence of external pH and fermentation products on ***Clostridium acetobutylicum*** intracellular pH and cellular distribution of fermentation products.. Appl Environ Microbiol.

[37] Madigan M, Martinko J (2005). Brock Biology of Microorganisms.

[38] Vogels G, Van der Drift C (1976). Degradation of purines and pyrimidines by microorganisms.. Microbiol Mol Biol Rev.

[39] Köpke M, Held C, Hujer S, Liesegang H, Wiezer A (2010). ***Clostridium ljungdahlii*** represents a microbial production platform based on syngas.. Proc Natl Acad Sci U S A.

[40] Durre P, Andreesen J (1983). Purine and glycine metabolism by ***Purinolytic clostridia***.. J Bacteriol.

[41] Lu C (2006). Pathways and regulation of bacterial arginine metabolism and perspectives for obtaining arginine overproducing strains.. Appl Microbiol Biotechnol.

[42] Pérez-Arellano I, Carmona-A_lvarez F, Gallego J, Cervera J (2010). Molecular mechanisms modulating glutamate kinase activity. Identification of the proline feedback inhibitor binding site.. J Mol Biol.

[43] Gamper H, Moses V (1974). Enzyme organization in the proline biosynthetic pathway of ***Escherichia coli***.. Biochimica et Biophysica Acta (BBA) - General Subjects.

[44] Chen L, Maloy S (1991). Regulation of proline utilization in enteric bacteria: Cloning and characterization of the ***Klebsiella*** put control region.. J Bacteriol.

[45] Milner J, McClellan D, Wood J (1987). Factors reducing and promoting the effectiveness of proline as an osmoprotectant in ***Escherichia*** coli K12.. J Gen Microbiol.

[46] Dand Vitreschak RodionovA, Aand Gelfand MironovM (2004). Comparative genomics of the methionine metabolism in gram-positive bacteria: A variety of regulatory systems.. Nucleic Acids Res.

[47] Andre G, Haudecoeur E, Monot M, Ohtani K, Shimizu T (2010). Global regulation of gene expression in response to cysteine availability in ***Clostridium perfringens***.. BMC Microbiol.

[48] Masip L, Veeravalli K, Georgiou G (2006). The many faces of glutathione in bacteria.. Antioxid Redox Signal.

[49] Nicolet Y, Fontecilla-Camps J, Fontecave M (2010). Maturation of [FeFe]-hydrogenases: Structures and mechanisms.. Int J Hydrogen Energy.

[50] Vignais P, Band Meyer BilloudJ (2001). Classification and phylogeny of hydrogenases.. FEMS Microbiol Rev.

[51] Peters J, Fisher K, Dean D (1995). Nitrogenase structure and function: A biochemical-genetic perspective.. Annu Rev Microbiol.

[52] Rey F, Oda Y, Harwood C (2006). Regulation of uptake hydrogenase and effects of hydrogen utilization on gene expression in ***Rhodopseudomonas palustris***.. J Bacteriol.

[53] Chen J, Toth J, Kasap M (2001). Nitrogen-fixation genes and nitrogenase activity in ***Clostridium acetobutylicum*** and ***Clsotridium beijerinckii***.. J Ind Microbiol Biotechnol.

[54] Foster J, Storz G, Hengge-Aronis R (2000). Microbial response to acid stress.. Bacterial Stress Responses.

[55] Foster J (2004). ***Escherichia coli*** acid resistance: Tales of an amateur acidophile.. Nat Rev Microbiol.

[56] Borden J, Sand Indurthi JonesD, Chen Y, Papoutsakis E (2010). A genomic-library based discovery of a novel, possibly synthetic, acid-tolerance mechanism in ***Clostridium acetobutylicum*** involving non-coding RNAs and ribosomal RNA processing.. Metab Eng.

[57] Gaudu P, Yamamoto Y, Pand Hammer JensenK, Gruss A, Fischetti VA, Novick RP, Ferretti JJ, Portnov DA, Rood JI (2006). Genetics of ***Lactococci***.. Gram-Positive Pathogens.

[58] Koyutürk M (2010). Algorithmic and analytical methods in network biology.. Wiley Interdiscip Rev Syst Biol Med.

[59] Lemosa R, Fernandesa A, Pereiraa M, Gomesa C, Teixeira M (2002). Quinol:fumarate oxidoreductases and succinate:quinone oxidoreductases: Phylogenetic relationships, metal centres and membrane attachment.. Biochim Biophys Acta.

[60] Thiele J, Zeikus J (1988). Control of interspecies electron ow during anaerobic digestion: Significance of formate transfer versus hydrogen transfer during syntrophic methanogenesis in ocs.. Appl Environ Microbiol.

[61] White D (2000). The physiology and biochemistry of prokaryotes.

[62] Mathews J, Wang G (2009). Metabolic pathway engineering for enhanced biohydrogen production.. Int J Hydrogen Energy.

[63] Koyuturk M, Kim Y, Subramaniam S, Szpankowski W, Grama A (2006). Detecting conserved interaction patterns in biological networks.. J Comput Biol.

[64] Tamura M, D'haeseleer P (2008). Microbial genotype-phenotype mapping by class association rule mining.. Bioinformatics.

[65] Liu Y, Li J, Sam L, Goh C, Gerstein M (2006). An integrative genomic approach to uncover molecular mechanisms of prokaryotic traits.. PLoS Comput Biol.

[66] Schmidt M (2010). Scalable Graph-Mining Techniques with Applications to Systems Biology [Ph.D. thesis].

[67] Koyutürk M, Grama A, Szpankowski W (2004). An efficient algorithm for detecting frequent subgraphs in biological networks.. Bioinformatics.

[68] Webb EC (1992). Enzyme Nomenclature 1992: Recommendations of the NCIUBMB on the Nomenclature and Classification of Enzymes.

[69] Gabi K, Maria S, Johann G, Hans-Werner M (2009). Uncovering metabolic pathways relevant to phenotypic traits of microbial genomes.. Genome Biol.

