# Supplementary material for: NIBBS-Search for Fast and Accurate Prediction of Phenotype-Biased Metabolic Systems
Source: PLoS Comput Biol. 2012 May 10;8(5):e1002490. doi: 10.1371/journal.pcbi.1002490 (PMC3349732; doi:10.1371/journal.pcbi.1002490)
Supplement: Table S14 — Enzymes related to acid-tolerent phenotype. This file consists of a comparison between enzymes in acid-tolerant organisms and alkaliphilic (non-acid-tolerant) organisms, in acid-tolerant experiments. Each row represents enzymes identified by NIBBS-Search and their corresponding pathways they are present in. The results are discussed in section Acid-tolerant Enzymes. (PDF) [file pcbi.1002490.s014.pdf]

Table 4 - Comparison between the presence (+) or absence (-) of enzymes in acid-tolerant organisms and alkaliphilic (non-acid-tolerant organisms), in acid-tolerant experiments. Each row represents enzymes identified by NIBBS and their corresponding pathways. Acid-tolerant organisms: *Clostridium acetobutylicum* **(cac)**, *Clostridium beijerinckii* **(cbe)**, *Clostridium perfringens* **(cpf)**, *Lactobacillus casei* **(lca)**, *Lactobacillus plantarum* **JDM1 (lpj)**, *Lactobacillus plantarum* **WCFS1 (lpl)**, *Streptococcus mutans* **(smu)**, *Gluconacetobacter diazotrophicus* **(gdj)**, *Pediococcus pentosaceus* **(ppe)**. **Non-acid-tolerant (alkaliphiles) included:** *Bacillus halodurans* **(bha)**, *Desulfurivibrio alkaliphilus* **(dak)**,*Ocenobacillus iheyensis* **(oih)**,*Bacillus pseudofirmus* **(bpf)**, *Alkaliphilus metalliredigens* **(amt)**, *Alkaliphlius oremlandii* **(aoe)**, *Alkalilimnicola ehrlichei (aeh), Bacillus clausii (bcl).*

EC Number	Enzyme Name	Pathway Name	Organisms																	
			Acid-tolerent									Alkaliphiles								
			cac	cbe	cpf	lca	lpj	lpl	lme	smu	gdj	ppe	bha	dak	oih	bpf	amt	aoe	aeh	bcl
3.5.1.14	aminoacylase	Arginine and proline metabolism	+	+	–	+	–	+	+	–	–	–	–	–	–	–	+	–	–	+
3.5.4.4	adenosine deaminase	Purine metabolism	+	–	+	–		+	+	+	–	–	–	–	–	–	–	–	–	–
4.1.1.15	glutamate decarboxylase	Taurine and hypotaurine metabolism	–	–	+	–	+	+	–	–	–	–	–	–	–	–	–	–	–	–
