# Supplementary material for: NIBBS-Search for Fast and Accurate Prediction of Phenotype-Biased Metabolic Systems
Source: PLoS Comput Biol. 2012 May 10;8(5):e1002490. doi: 10.1371/journal.pcbi.1002490 (PMC3349732; doi:10.1371/journal.pcbi.1002490)
Supplement: Table S16 — Enzymes related to TCA/rTCA expression. This file consists of a comparison, presence (+) or absence (−) of enzymes across the set of organisms used in the TCA and rTCA experiments. (PDF) [file pcbi.1002490.s016.pdf]

Table 7 - The presence (+) or absence (-) of enzymes in the organisms used in the TCA and rTCA experiments. rTCA organisms: *Chlorobaculum tepidum* **(cte)**, *Chlorobium limicola* **(cli)**, *Sulfurimonas denitrificans* **(tdn)**, *Aquifex aeolicus* **(aae)**, *Hydrogenobacter thermophilus* **(hth)**, *Nautilia profundicola* **(nam)**. **TCA organisms:** *Bordetella broniseptica* **(bbr)**, *Staphylococcus saprophyticus* **(ssp)**, *Myxococcus xanthus* **(mxa)**, *Leptospira interrogans serovar lai* **(lil)**, *Helicobacter pylori* **(hpa)**, *Listeria innocua* **(lin)**, *Escherichia coli* **(eco)**, *Shewanella oneidensis* **(son)**,*Anaplasma marginale St. Maries* **(ama)**,*Bdellovibrio bacteriovorus* **(bba)**, *Bordetella parapertussis* **(bpa)**, *Bordetalla bronchiseptica* **(bbr)**,*Geobacillus kaustophilus* **(gka)**, *Legionella pneumophila Lens* **(lpf)**,*Neisseria gonorrhoeae* **(ngo)**, *Sinorhizobium meliloti* **(sme)**.

EC Number	Enzyme Name	rTCA organisms						TCA organisms															
		cte	cli	tdn	aae	hth	nam	bbr	ssp	mxa	lil	hpa	lin	eco	son	ama	bba	bpa	bbr	gka	lpf	ngo	sme
**Common Enzymes**																							
4.2.1.3	aconitase	+	+	+	+	+	+	+	+	+	+	+	+	+	+	+	+	+	+	+	+	+	+
1.1.1.42	isocitrate dehydrogenase	+	+	+	+	+	+	+	+	+	+	+	+	+	+	+	+	+	+	−	+	+	+
6.2.1.5	succinate thiokinase	+	+	+	+	+	+	+	+	+	+	−	−	+	+	+	+	+	+	+	+	+	+
4.2.1.2	fumarase	+	+	+	+	+	+	+	+	+	+	+	+	+	+	+	+	+	+	+	+	+	+
1.1.1.37	malate dehydrogenase	+	+	+	+	+	+	+	+	+	+	−	−	+	+	+	+	+	+	+	+	−	+
**rTCA Specific Enzymes**																							
1.2.7.3	2-oxoglutarate synthase	+	+	+	−	+	+	−	+	−	−	+	−	−	−	−	−	−	−	−	−	−	−
2.3.3.8	ATP citrate synthase	+	+	+	−	−	+	−	−	−	−	−	−	−	−	−	−	−	−	−	−	−	−
1.3.1.6	fumarate reductase	−	−	−	−	−	−	−	−	−	−	−	−	−	−	−	−	−	−	−	−	−	−
**TCA Specific Enzymes**																							
2.3.3.1	citrate (Si)-synthase	+	+	+	+	+	−	+	+	+	+	+	+	+	+	+	+	+	+	+	+	+	+
1.2.4.2	alpha ketoglutarate dehydrogenase	−	+	−	−	−	−	+	+	+	+	−	−	+	+	+	+	+	+	+	+	+	+
1.3.99.1	succinate dehydrogenase	+	+	+	+	+	+	+	+	+	+	+	+	+	+	+	+	+	+	+	+	+	+
