# Supplementary material for: NIBBS-Search for Fast and Accurate Prediction of Phenotype-Biased Metabolic Systems
Source: PLoS Comput Biol. 2012 May 10;8(5):e1002490. doi: 10.1371/journal.pcbi.1002490 (PMC3349732; doi:10.1371/journal.pcbi.1002490)
Supplement: Text S3 — NIBBS-Search Algorithm Pseudocode. This file contains more details on the NIBBS-Search algorithm. (PDF) [file pcbi.1002490.s024.pdf]

NIBBS-Search Algorithms Pseudocode

Algorithm 1: NIBBS-Search: Network Instance Based Biased Subgraph Search

Input: P—A set of positive network instances
N—A set of negative network instances
ϕ_0—The minimum bias score for seed expansion
Output: M—A set of maximally-biased subgraphs

$\Gamma \leftarrow$ `GenerateSeedSets()`;
Remove all seed sets S from Γ where $\phi(S, P, N) < \phi_0$;
foreach *seed set* $S \in \Gamma$ **do**
    $M = M \cup$ `ExpandSeedSet()`;

Algorithm 2: GenerateSeedSets

Input: E_G—The edge set of the network map
κ—The maximum size of the seed set
Output: R—A set of seed sets

$E' \leftarrow$ Sort the edges in E_G by their ϕ-values;
while E' *contains unmarked edges* **do**
    $e_0 = \leftarrow$ Unmarked edge in E' with the least ϕ-value;
    $S \leftarrow S \cup e_0$;
    Mark e_0;
    $C \leftarrow$ `GenerateSeedCandidates(`S`)` ;
    while $C \neq \emptyset$ *AND* $|S| \leq \kappa$ **do**
        $e \leftarrow$ Best candidate in C;
        $S \leftarrow S \cup e$;
        Mark e;
        $C \leftarrow$ `GenerateSeedCandidates(`S`)`
    $R \leftarrow R \cup S$;

Algorithm 3: ExpandSeedSet

Input:
S—A seed set of edges
α—Expansion bound
Output: S—The expanded set of edges

$C \leftarrow$ `GenerateExpansionCandidates(`S`, `α`)`;
while $C \neq \emptyset$ **do**
  $e \leftarrow$ Select best candidate from C;
  $S \leftarrow S \cup e$;
  $C \leftarrow$ `GenerateExpansionCandidates(`S`, `α`)`;
